# Comparative genomic analysis identifies X-factor (haemin)-independent *Haemophilus haemolyticus*: a formal re-classification of '*Haemophilus intermedius*'

**DOI:** 10.1099/mgen.0.000303

**Published:** 2019-12-20

**Authors:** Tegan M. Harris, Erin P. Price, Derek S. Sarovich, Niels Nørskov-Lauritsen, Jemima Beissbarth, Anne B. Chang, Heidi C. Smith-Vaughan

**Affiliations:** ^1^​ Child Health Division, Menzies School of Health Research, Darwin, NT, Australia; ^2^​ GeneCology Research Centre, University of the Sunshine Coast, Sippy Downs, QLD, Australia; ^3^​ Department of Clinical Microbiology, Aarhus University Hospital, Aarhus, Denmark; ^4^​ Department of Respiratory and Sleep Medicine, Queensland Children’s Hospital, Brisbane, QLD, Australia; ^5^​ School of Medicine, Griffith University, Gold Coast, QLD, Australia

**Keywords:** *Haemophilus haemolyticus*, haemin-independent *Haemophilus haemolyticus*, *'Haemophilus intermedius'*, haemin biosynthesis, comparative genomics

## Abstract

The heterogeneous and highly recombinogenic genus *Haemophilus* comprises several species, some of which are pathogenic to humans. All share an absolute requirement for blood-derived factors during growth. Certain species, such as the pathogen *Haemophilus influenzae* and the commensal *Haemophilus haemolyticus *, are thought to require both haemin (X-factor) and nicotinamide adenine dinucleotide (NAD, V-factor), whereas others, such as the informally classified ‘*Haemophilus intermedius* subsp. *intermedius*’, and *Haemophilus parainfluenzae *, only require V-factor. These differing growth requirements are commonly used for species differentiation, although a number of studies are now revealing issues with this approach. Here, we perform large-scale phylogenomics of 240 *Haemophilus* spp. genomes, including five ‘*H. intermedius*’ genomes generated in the current study, to reveal that strains of the ‘*H. intermedius*’ group are in fact haemin-independent *H. haemolyticus* (hi*Hh*). Closer examination of these hi*Hh* strains revealed that they encode an intact haemin biosynthesis pathway, unlike haemin-dependent *H. haemolyticus* and *H. influenzae *, which lack most haemin biosynthesis genes. Our results suggest that the common ancestor of modern-day *H. haemolyticus* and *H. influenzae* lost key haemin biosynthesis loci, likely as a consequence of specialized adaptation to otorhinolaryngeal and respiratory niches during their divergence from *H. parainfluenzae *. Genetic similarity analysis demonstrated that the haemin biosynthesis loci acquired in the hi*Hh* lineage were likely laterally transferred from a *H. parainfluenzae* ancestor, and that this event probably occurred only once in hi*Hh*. This study further challenges the validity of phenotypic methods for differentiating among *Haemophilus* species, and highlights the need for whole-genome sequencing for accurate characterization of species within this taxonomically challenging genus.

## Data Summary

Illumina NextSeq 500 whole-genome sequencing data generated from five ‘*Haemophilus intermedius* subsp. *intermedius*’ are available as 150 bp paired-end reads from the National Center for Biotechnology Information sequence read archive (SRA) under BioProject PRJNA509094. Whole-genome sequencing data for an additional 42 *Haemophilus* *haemolyticus* (including 6 haemin-independent strains), generated as part of previous genomic studies within our laboratory, have also been made available under BioProject PRJNA509094 as Illumina HiSeq 100 bp paired-end reads. Additionally, draft genome assemblies of the 11 haemin-independent *H. haemolyticus* are available from GenBank. The SRA and GenBank accession numbers are listed in Table S1 (available with the online version of this article). Accession numbers for publicly available *Haemophilus influenzae *, *H. haemolyticus *, *Haemophilus parainfluenzae* and *Haemophilus* spp. genomes used in this study are summarized in Table S1.

**Impact Statement**

The human pathogen *Haemophilus influenzae *, and the closely related *Haemophilus haemolyticus *, a commensal of the human upper respiratory tract, require an exogenous source of the blood-derived factors haemin and nicotinamide adenine dinucleotide (NAD) for growth. Dependence on haemin and NAD is the primary phenotype used to discriminate these two species from other *Haemophilus* species, such as *Haemophilus parainfluenzae *, which requires only NAD supplementation for growth. Using comparative genomics, we assigned strains to a new lineage of *H. haemolyticus* that can synthesize haemin, a novel phenotype for this species. Herein termed haemin-independent *H. haemolyticus* (hi*Hh*), members of this lineage harbour the complete set of the genes that encode a functional haemin biosynthesis pathway. We further demonstrated that members of the informal species ‘*Haemophilus intermedius*’ also reside in the hi*Hh* lineage, resulting in a formal reclassification of this previously ‘fuzzy’ *Haemophilus* species. This work highlights the heterogeneous nature of *Haemophilus* genomes, and further demonstrates that accurate characterization of *Haemophilus* species cannot be achieved from phenotypic characteristics alone.

## Introduction

The genus *Haemophilus* currently comprises 14 species that have been formally classified, 9 of which demonstrate host specificity for humans [1]. Additional informal *Haemophilus* species (e.g. ‘*Haemophilus intermedius*’) have also been described in the literature [2, 3]. All species have an absolute growth requirement for haemin (X-factor) and/or nicotinamide adenine dinucleotide (NAD, V-factor), both of which are derived from blood [1, 4]. The production of catalase, β-galactosidase, tryptophanase, urease, ornithine decarboxylase and haemolysis are additional phenotypic attributes used to characterize *Haemophilus* spp. [1]. However, such phenotypes can be variable both within and between species [1], resulting in ample opportunity for species misidentification.

Among the *Haemophilus* spp., *Haemophilus influenzae* is considered to be the most clinically relevant (especially for invasive disease), and much effort has been applied to its discrimination from other *Haemophilus* species. X- and V-factor dependence is the primary phenotypic method used to discriminate *H. influenzae* and *Haemophilus haemolyticus* from *Haemophilus parainfluenzae* in diagnostic and clinical trial settings [5, 6]. However, discrimination of *H. influenzae* from *H. haemolyticus* is more difficult. Both species occupy the same environmental niche, are thought to require both X- and V-factor for growth, and non-haemolytic *H. haemolyticus* strains can be morphologically indistinguishable from non-typeable *H. influenzae* [7]. Due to shared phenotypic characteristics between non-typeable *H. influenzae* and non-haemolytic *H. haemolyticus *, rapid (API NH; bioMeriéux) and automated biochemical differentiation [MALDI-TOF MS methods, such as VITEK 2 NH (bioMeriéux)] are also problematic, with false positive rates of up to 10 % [8–11] and species misidentification reported [11]. The close similarity of *H. influenzae* and *H. haemolyticus* also extends to a genetic level, with frequent recombination within and between these species [12, 13], particularly in non-typeable *H. influenzae* [14]. Hence, molecular discrimination of these species using PCR and fluorescence *in situ* hybridization (FISH) approaches have also been challenging [15, 16]. Thus, the availability of large-scale genomic data has been essential for correct phylogenetic placement of *Haemophilus* species and the identification of species-specific molecular targets to differentiate these two highly related but distinct species [17–21].

To further challenge our understanding of the characteristics used to delineate *Haemophilus* species, a haemin-synthesizing lineage of *Haemophilus* that is closely related to *H. influenzae* and *H. haemolyticus *, yet does not require haemin for growth, was identified in 1987 [2, 3]. Informally referred to as ‘*Haemophilus intermedius* subsp. *intermedius*’ or more simply, ‘*Haemophilus intermedius*’, these strains demonstrated similarity to *H. influenzae* using DNA–DNA hybridization [2]. However, in addition to only requiring V-factor for growth, their ability to ferment sucrose conflicted with key *H. influenzae* phenotypes [2]. In an attempt to delineate *H. influenzae* species boundaries, Nørskov-Lauritsen and colleagues further investigated difficult-to-classify *Haemophilus* spp., including the haemin-synthesizing ‘*H. intermedius*’ [3]. They observed that sucrose fermentation and haemin biosynthesis only ever occurred together, and phylogenetic relationships inferred from housekeeping gene and 16S rDNA sequences demonstrated that haemin-synthesizing strains fell outside the *H. influenzae* cluster, indicating that, whilst closely related, these strains were not *H. influenzae *. Further investigation of this unusual lineage showed the presence of chromosomally encoded haemin biosynthesis genes; however, these genes had no evidence of recent transfer from *H. parainfluenzae *, suggesting a more ancestral origin [3]. The evolutionary dynamics of this unusual *H. influenzae *-like, haemin-independent lineage has remained enigmatic.

To better understand the genetic relatedness of *H. influenzae* and *H. haemolyticus* near-neighbour species, six suspected *H. parainfluenzae* (which requires only V-factor for growth [1]) were genome sequenced. Comparative genomics demonstrated these isolates were highly genetically similar to *H. haemolyticus *, indicating that these isolates were related to the haemin-synthesizing ‘*H. intermedius*’. Here, we used comparative genomic analyses to reconstruct a phylogeny of 240 *H *. *influenzae *, *H. haemolyticus* and *H. parainfluenzae* strains to determine the phylogenomic placement of ‘*H. intermedius*’ among the established *Haemophilus* species clades. We subsequently investigated the genomes of 14 haemin-independent isolates previously identified as ‘*H. intermedius*’, *H. haemolyticus* or undefined *Haemophilus* spp. for the presence of haemin biosynthesis genes to genetically confirm the ability of these strains to grow in the absence of haemin, and to investigate the diversity and origin of these gene pathways in this unusual clade.

## Methods

### *Haemophilus* genomes

In total, 240 *Haemophilus* spp. genomes were examined in this study (Table S1). Forty-five *H. haemolyticus* (including six haemin-independent isolates) and three *H. parainfluenzae* genomes were generated as part of previous genomics studies within our laboratory [17, 18]. In the current study, we generated genome sequence data for five previously reported haemin-independent ‘*H. intermedius*’ strains (CCUG 11096, CCUG 15949, CCUG 30218, CCUG 31732, PN24 [3]; Table S1). DNA was extracted using a DNeasy blood and tissue kit (Qiagen) and diluted to 0.30 ng µl^−1^. DNA libraries were prepared from 1 ng genomic DNA on a Janus NGS Express robot (Perkin Elmer), using the Nextera XT DNA sample preparation kit in combination with the Nextera XT Index kit v2, set D (Illumina) according to the manufacturers' protocols. Dual-indexed paired-end 150 bp sequencing was performed on the Illumina NextSeq 500 using v2 chemistry on a medium flow cell (Illumina). We included publicly available genomes for an additional 152 *H *. *influenzae *, 12 *H *. *haemolyticus *, 21 *H *. *parainfluenzae* and 2 *Haemophilus* spp. (CCUG 66565 and F0629) [22–36] (Table S1). Previously incorrect [24] or incomplete species designations for 839_HINF, C1, F0397, 137_HINF, 159_HINF, 167_HINF, 614_HPAR and 841_HINF were changed based on our prior phylogenomic analyses [18], and the *Haemophilus* spp. strains CCUG 66565 and F0629 were reassigned to haemin-independent *H. haemolyticus* (hi*Hh*) based on the phylogenomic analysis performed in the current study. In total, 64 *H *. *haemolyticus* genomes were used in this study, including 14 hi*Hh*/‘*H. intermedius*’ isolates. Details for these hi*Hh*/‘*H. intermedius*’ isolates are listed in Table 1.

**Table 1.** hi*Hh* isolates used in this study

Isolate	Anatomical site	Country of origin	Year of isolation	Haemin (X-factor) dependence*	NAD (V-factor) dependence*	Haemin biosynthesis pathway†	Genome reference
60819_B_Hi1	BAL	Australia	2010	−	+	C_5_, PP-dependent, O_2_-independent	[18]
60824_B_Hi4	BAL	Australia	2010	−	+	C_5_, PP-dependent, O_2_-independent	[18]
60971_B_Hi3	BAL	Australia	2012	−	+	C_5_, PP-dependent, O_2_-independent	[18]
60982_B_Hi1	BAL	Australia	2012	−	+	C_5_, PP-dependent, O_2_-independent	[18]
65117_B_Hi3	BAL	Australia	2011	−	+	C_5_, PP-dependent, O_2_-independent	[18]
65151_B_Hi4	BAL	Australia	2011	−	+	C_5_, PP-dependent, O_2_-independent	[18]
839_HINF	BAL	USA	2013	Unknown	Unknown	C_5_, PP-dependent, O_2_-independent	[24]
CCUG 11096	Pleural fluid	Sweden	1981	−	+	C_5_, PP-dependent, O_2_-independent	[3]
CCUG 15949	Eye	Sweden	1984	−	+	C_5_, PP-dependent, O_2_-independent	[3]
CCUG 30218	Cerebrospinal fluid	Sweden	1992	−	+	C_5_, PP-dependent, O_2_-independent	[3]
CCUG 31732	Ascitic fluid	Sweden	1993	−	+	C_5_, PP-dependent, O_2_-independent	[3]
CCUG 66565	Sputum	Sweden	2014	Unknown	Unknown	C_5_, PP-dependent, O_2_-independent	This study
F0629	Oral cavity	USA	2015	Unknown	Unknown	C_5_, PP-dependent, O_2_-independent	This study
PN24	Urine	Denmark	2004	−	+	C_5_, PP-dependent, O_2_-independent	[3]

BAL, Bronchoalveolar lavage.

*, Recorded phenotype.

†, Aminolevulinic acid biosynthesis occurs using the C_5_ pathway [63]; coproporphyrinogen III conversion to protohaem is protoporphyrin-dependent, and occurs in an oxygen-independent manner [63].

### Genome assemblies

Reference-assisted genome assemblies of previously unassembled *Haemophilus* genomes were generated with the Microbial Genome Assembler Pipeline (mgap) v0.0.1 (https://github.com/dsarov/MGAP---Microbial-Genome-Assembler-Pipeline) [37], which wraps Trimmomatic [38], Velvet [39], Gapfiller [40], abacas [41], image [42], sspace [43] and icorn2 [44], using default parameters. For assembling ‘*H. intermedius*’ genomes, the single-contig assembly of *H. haemolyticus* NCTC 10839 (GenBank accession no. LS483458.1) was used as the reference sequence. Species classification was based on phylogenomic grouping [17, 18, 36]. Species designation of apparent *H. haemolyticus* genomes (including the 14 hi*Hh*/‘*H. intermedius*’) was confirmed by *in silico* detection of the *H. haemolyticus* molecular target *hypD* and the absence of the *H. influenzae* molecular target *siaT* [18]. Genome assemblies were annotated using Prokka v1.12-beta [45] with the --usegenus flag.

### Phylogenetic analysis

To reconstruct a phylogeny of *Haemophilus* spp., sequence data for the 240 *Haemophilus* spp. genomes were mapped against the complete genome of *H. influenzae* 86–028 NP (GenBank accession no. CP000057.2) using SPANDx v3.2.1 [46], a genomics pipeline for comparative analysis of haploid genome datasets, which wraps Burrows-Wheeler Aligner [47], SAMtools [48], Picard Tools and Genome Analysis Tool Kit [49]. A *H. haemolyticus* phylogeny was also generated, where the 64 *H *. *haemolyticus* genomes were mapped to the merged, multi-contig assembly of the hi*Hh* strain 60819_B_Hi1 (GenBank accession no. SDPA00000000) as the reference. Maximum parsimony phylogenomic trees were generated using paup v4.0a153 [50] and visualized using FigTree (http://tree.bio.ed.ac.uk/software/figtree/). Bootstrapping was performed in paup with 1000 replicates.

To confirm accurate phylogenetic placement of the 14 hi*Hh*, ‘*H. intermedius*’ and *Haemophilus* spp. strains, the average nucleotide identity (ANI) with reference to the *H. haemolyticus* NCTC 10839, non-typeable *H. influenzae* 86–028 NP and *H. parainfluenzae* T3T1 genomes was calculated using fastANI [51] with default parameters.

### Haemin biosynthesis pathway gene identification

Translated haemin biosynthesis pathway gene sequences (*hemA*, *PARA_RS02505*; *hemL*, *PARA_RS08795*; *hemB*, *PARA_RS01040*; *hemC*, *PARA_RS02495*; *hemD*, *PARA_RS02500*; *hemE*, *PARA_RS04400*; *hemN*, *PARA_RS04215*; *hemG*, *PARA_RS02230*; *hemH*, *PARA_RS09990*) were extracted from the T3T1 *H. parainfluenzae* genome (GenBank accession no. NC_015964.1), and queried against a database containing the 64 *H *. *haemolyticus* genomes using tBLASTn (blast+ v.2.2.29) [52]. Genome assembly annotations were manually reviewed to confirm the presence of haemin biosynthesis genes.

To optimize assembly of genetic regions harbouring haemin biosynthesis genes for downstream analysis, mgap assemblies were repeated using the hi*Hh* 60819_B_Hi1 assembly as the scaffolding reference for the remaining 13 hi*Hh*/‘*H. intermedius*’ strains, and 9 haemin-dependent *H. haemolyticus* (hd*Hh*) strains. For each assembly, contigs were rearranged with car [53] using 60819_B_Hi1 as the reference prior to merging into a single contig. Genome assemblies were annotated using Prokka v1.12-beta [45]. Locally collinear block analyses of the annotated genome assemblies were performed using progressiveMAUVE v20150226 build 10 [54]. Genome alignments were visually assessed to determine whether nucleotide regions encoding haemin biosynthesis genes were syntenic. Artemis comparison tool (act) v13.0.0 [55] was used to visually represent the genetic architecture surrounding the haemin biosynthesis genes in *H. haemolyticus *. act plots were generated by comparing assembled whole genomes of representative ‘*H. intermedius*’ and *H. haemolyticus* strains to the 60819_B_Hi1 reference. To ensure that assembly scaffolding did not bias the results, the progressiveMAUVE analysis was repeated using mgap *de novo* assemblies (generated using default parameters).

### Haemin biosynthesis pathway gene acquisition

In 2009, Nørskov-Lauritsen and colleagues showed that three haemin biosynthesis loci in ‘*H. intermedius*’ strains (*hemB*, *hemE*, *hemN*) appeared to be ancestral, with no evidence of recent lateral transfer from, for example, *H. parainfluenzae* [3]. To determine whether haemin biosynthesis pathway genes have evolved similarly to whole-genome evolution in hi*Hh*/‘*H. intermedius*’, hi*Hh*/‘*H. intermedius*’ Illumina data were aligned to a concatenated nucleotide sequence of haemin biosynthesis gene sequences extracted from the 60819_B_Hi1 assembly, or the merged, multi-contig assembly of 60819_B_Hi1, using SPANDx v.3.2.1. Maximum parsimony phylogenies were reconstructed from the orthologous SNP matrices and bootstrapped as described above. Phylogenies were compared by plotting a tanglegram in Dendroscope v.3.5.10 [56].

To confirm hi*Hh*/‘*H. intermedius*’ haemin biosynthesis genes were not recently acquired from *H. parainfluenzae *, the concatenated nucleotide sequences of the *H. parainfluenzae* T3T1 haemin biosynthesis genes were used as the reference for a SPANDx alignment of the 14 hi*Hh*/‘*H. intermedius*’ genomes and 24 *H *. *parainfluenzae* genomes. A maximum-likelihood phylogenetic tree was generated using RAxML [57].

To measure selective pressures on haemin biosynthesis gene maintenance, the ratio of non-synonymous to synonymous SNPs (dN/dS) in haemin biosynthesis genes was determined for the 14 hi*Hh*/‘*H. intermedius*’ and 24 *H *. *parainfluenzae* strains. dN/dS ratios were calculated from multi-fasta files of *hem* gene sequences extracted from genome assemblies using slac [58] via the Datamonkey web application [59]. To compare, dN/dS ratios were also determined for the *H. influenzae* MLST genes *adk*, *atpG*, *frdB*, *mdh*, *pgi* and *recA* in the 14 hi*Hh*/‘*H. intermedius*’ strains.

To determine the unique genetic content of the 14 hi*Hh*/‘*H. intermedius*’ when compared with hd*Hh*, a pangenome of the 64 *H *. *haemolyticus* genomes was generated using Roary v.3.12.0 [60], with an amino acid percentage identity cut-off of 85 %. A cut-off lower than the default (95 %) was used to reduce false classification of core genes shared by all *H. haemolyticus* as accessory genes due to potential sequence variation within the hi*Hh*/‘*H. intermedius*’ genomes relative to hd*Hh*. The pangenome was interrogated using plink v.1.07 [61] and the GeneratePLINK_Roary.sh script distributed within the SPANDx package [46] for the retrieval of coding sequences unique to either the 14 hi*Hh*/‘*H. intermedius*’ or the 50 hd*Hh* genomes. These unique genes were compared to the closed genome of *H. parainfluenzae* T3T1 using large-scale blast score ratio (ls-bsr, v1.00) [62] to ascertain their presence in this species. A blast score ratio (BSR) of ≥0.8 was considered indicative of potential acquisition due to recombination with *H. parainfluenzae *.

## Results

### Phylogenomics confirms that '*H. intermedius*' is in fact hi*Hh*

**Fig. 1.**
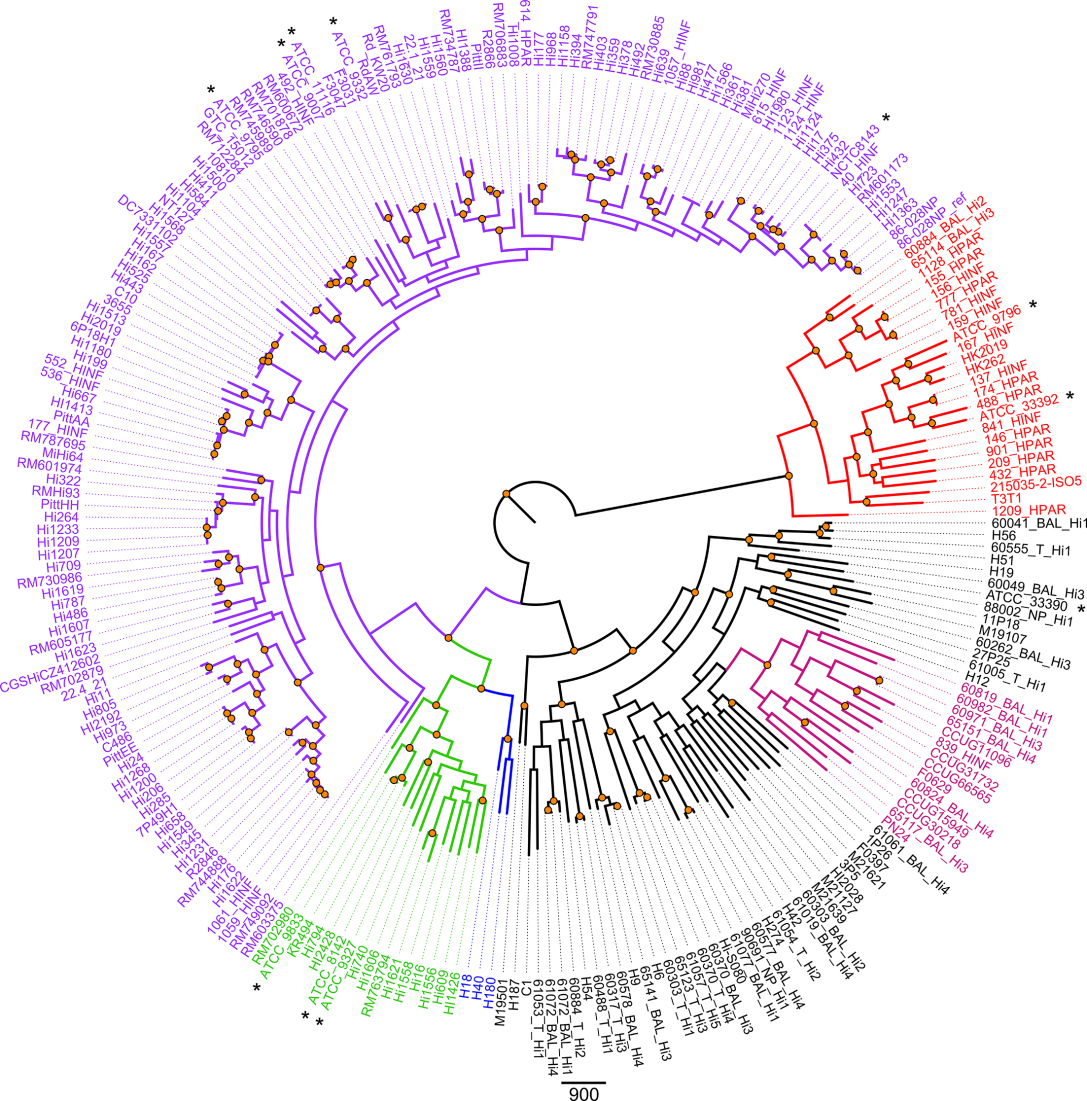
Phylogenomic analysis of 240 *Haemophilus* spp. to identify placement of hi*Hh* and ‘*H. intermedius*’. A midpoint-rooted maximum parsimony tree was constructed using 30 345 orthologous, biallelic SNPs found among 152 *H *. *influenzae* (purple), including 16 clade I (green) [36] and three *fucP*-negative clade (blue) [17, 18] genomes, 64 *H *. *haemolyticus* (black) including 14 hi*Hh* and ‘*H. intermedius*’ genomes (pink), and 24 *H *. *parainfluenzae* genomes (red). Consistency index=0.1482. Bootstrap values were inferred from 1000 replicates. Clades with >80 % support are annotated with a filled orange circle. Type strains are denoted with an asterisk. Bar, 900 bp.

Phylogenomic reconstruction of 240 *Haemophilus* spp. genomes was carried out to determine the relatedness of the ‘*H. intermedius*’ isolates at the whole-genome level when compared with *H. influenzae *, *H. haemolyticus* and *H. parainfluenzae* strains. The six hi*Hh* strains described in this study, 839_HINF [24], the *Haemophilus* sp. strains CCUG 66565 and F0629, and five previously assigned ‘*H. intermedius*’ (Tables 1and S1) all share a recent common ancestor and form a subclade within the *H. haemolyticus* clade (Fig. 1). Bootstrapping demonstrated that the hi*Hh* subclade is 100 % supported (Fig. 1), and a maximum-likelihood phylogenomy verified the topology of the maximum parsimony phylogenomic reconstruction (Fig. S1). To confirm correct species association, the ANIs of the 64 genomes in the *H. haemolyticus* clade were calculated compared to NCTC 10839 *H *. *haemolyticus *, 86–028 NP non-typeable *H. influenzae* and T3T1 *H. parainfluenzae* genomes (Fig. S2). ‘*H. intermedius*’/hi*Hh* genomes demonstrated the highest ANI to *H. haemolyticus* (92.95–93.44%), followed by *H. influenzae* (82.90–90.32%) and *H. parainfluenzae* (81.34–81.94%). Higher ANIs were observed for the hd*Hh* genomes to *H. haemolyticus* (94.06–95.87%) and *H. influenzae* (91.21–92.85%), and a comparable ANI to *H. parainfluenzae* (81.02–82.37%) (Fig. S2). These results confirm that the informal ‘*H. intermedius*’ nomenclature should be renamed as hi*Hh* to more accurately reflect its species designation, whilst differentiating this unusual clade from conventional haemin-dependent strains.

### A complete haemin biosynthesis pathway is present in hi*Hh*

**Fig. 2.**
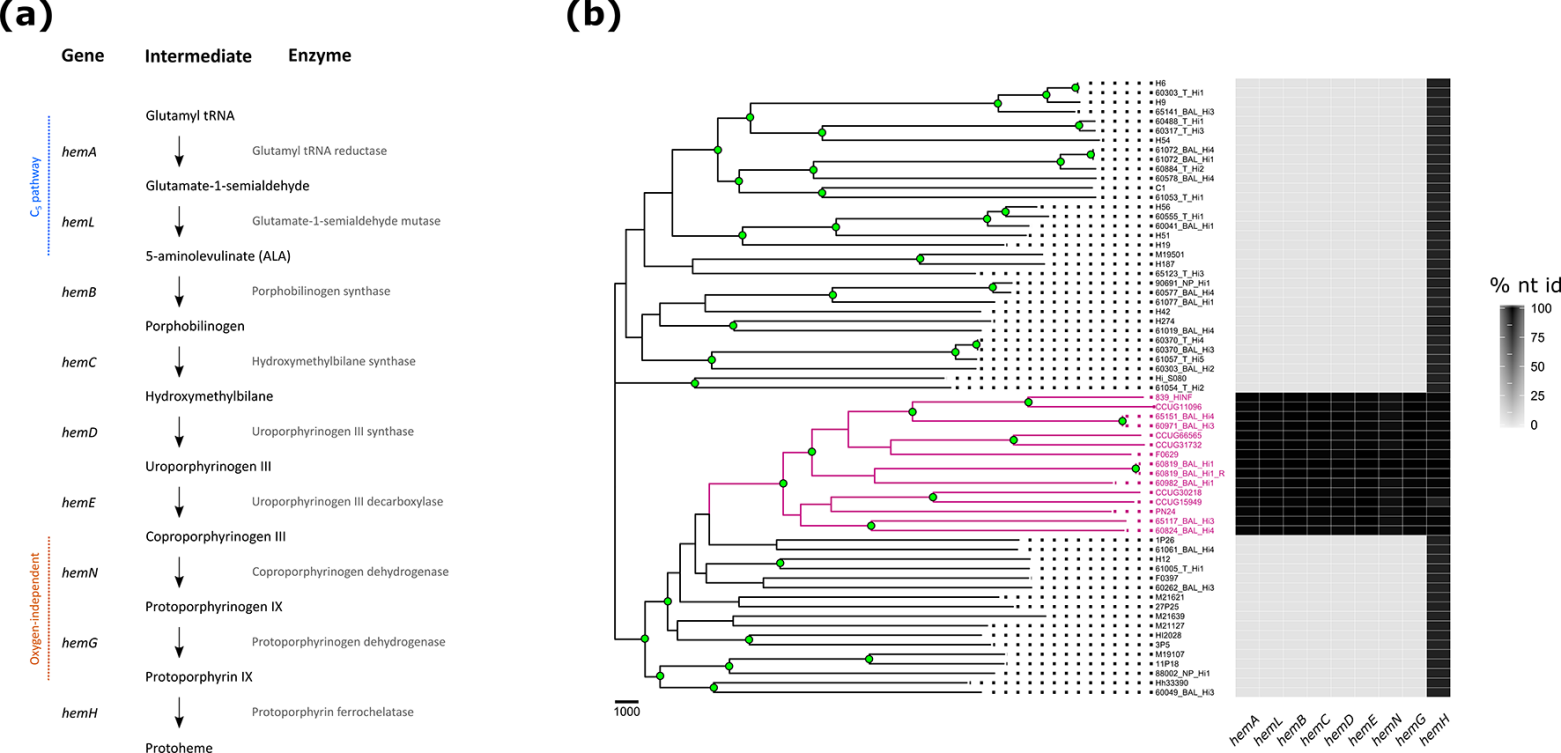
The haemin biosynthesis pathway in hi*Hh*. (a) The hi*Hh* strains synthesize haemin by utilizing the C_5_ pathway for 5-aminolevulinic acid synthesis. Conversion of coproporphyrinogen III to protohaem occurs in a protoporphyrin-dependent, oxygen-independent manner. (b) Heatmap showing the percentage nucleotide identity of haemin biosynthesis genes when compared to reference gene sequences extracted from the assembled genome of hi*Hh* 60819_BAL_Hi1. The heatmap is plotted against a *H. haemolyticus* midpoint-rooted, maximum parsimony tree, constructed using 153 468 orthologous, biallelic SNPs found amongst the 64 *H *. *haemolyticus* genomes. hi*Hh* are shown in pink. Consistency index=0.2380. Bootstrap values were inferred from 1000 replicates. Nodes with >80 % support are annotated with a filled green circle. Bar, 1000 bp.

Consistent with their less fastidious growth requirements, genes encoding a functional haemin biosynthesis pathway were identified in the genomes of all 14 hi*Hh* (Fig. 2). Based on their gene complement, these isolates utilize the C_5_ pathway of aminolevulinic acid (ALA) biosynthesis, as signified by genes encoding a Glu-tRNA reductase (*hemA*; *PARA_RS02505* in *H. parainfluenzae* T3T1) and a glutamate-1-semialdehyde mutase (*hemL*; *PARA_RS08795* in *H. parainfluenzae *) [63]. The conversion of coproporphyrinogen III to protohaem is protoporphyrin-dependent, and occurs in an oxygen-independent manner in these isolates (Fig. 2) [63], consistent with *H. parainfluenzae* haemin biosynthesis.

The hi*Hh* genomes harboured two genes annotated as oxygen-independent coproporphyrinogen III oxidases (*hemN*). The *hemN* paralogues demonstrated <32 % amino acid identity within each of the hi*Hh* genomes, indicating that they likely encode non-homologous isofunctional enzymes. However, further investigation of the *hemN* genes revealed that only one *hemN* was correctly annotated. The true *hemN* demonstrated 80 % amino acid identity to *PARA_RS04215*, which encodes an oxygen-independent coproporphyrinogen III oxidase in *H. parainfluenzae* T3T1. Further, a 67 % amino acid identity match to *hemN* of *Escherichia coli* (GenBank accession no. NC_000913.3) [64], and the occurrence of the two regions integral to HemN function (_18_GPRYTSYPTA_27_ and _306_RNFQGYTT_313_) demonstrated that the true hi*Hh hemN* gene encodes a functional coproporphyrinogen III oxidase [65].

The incorrectly annotated *hemN* gene demonstrated 87 % amino acid identity to the *H. parainfluenzae* T3T1 gene *PARA_RS03220*. This gene encodes the radical *S*-adenosyl methionine (SAM) family haem chaperone protein HemW, which is not part of the haemin biosynthesis pathway (Fig. 2a). The absence of the HemN functional regions and poor matching (29 % amino acid identity) to *E. coli hemN* is consistent with incorrect annotation of this gene [65].

The last gene in the protoporphyrin-dependent haemin-biosynthesis pathway, *hemH (PARA_RS09990*), encodes a protoporphyrin ferrochelatase that is ubiquitous in all *H. influenzae* [1] and *H. haemolyticus* genomes (Fig. 2b). *hemH* is likely a remnant of the original haemin biosynthesis pathway harboured by the *H. influenzae */* H. haemolyticus */* H. parainfluenzae* ancestor and, thus, is the only *hem* gene not reacquired by hi*Hh*. An additional gene associated with the haemin biosynthesis pathway, *hemX* (*PARA_RS02505*), was also identified in all 64 *H *. *haemolyticus* genomes. Encoding a uroporphyrinogen III methyltransferase, *hemX* is required for the conversion of uroporphyrinogen III to precorrin-2, the substrate required for sirohaem synthesis [66]. *hemX* was also observed in all 24 *H *. *parainfluenzae* genomes examined in this study.

### Each of the dispersed locations of haemin biosynthesis genes is syntenic across the 14 hi*Hh* genomes

The progressiveMAUVE analysis demonstrated that the *H. haemolyticus* genomes consist of a very high number of predicted syntenic blocks, which are much smaller in size than the assembled contigs, and whose order is not very conserved. The haemin biosynthesis pathway genes are not found within a single operon on the hi*Hh* chromosome; rather, the eight loci are located in seven distinct regions across the genome. The exception is the *hemCD* cluster (*PARA_RS02495* and *PARA_RS02500*, respectively), which occurs in a ~5 kbp syntenic block in all 14 hi*Hh* genomes, commencing with *hemC* (Fig. S3); all other core *hem* loci in the hi*Hh* strains are found within individual syntenic blocks. In hd*Hh* genomes, *hemC* and *hemD* are absent and the syntenic block instead commences with *hemX* (Fig. S3).

The *hemA* (~4.3 kbp), *hemB* (~2.7 kbp), *hemE* (~14.4 kbp) and *hemG* (~3.3–3.6 kbp) syntenic block structures are relatively well-conserved amongst the hi*Hh* genomes (Figs S4, S5, S6 and S7), and these loci are absent in hd*Hh* strains. *hemN* was the only haemin biosynthesis gene that did not reside in a syntenic block (Fig. S8). In the progressiveMAUVE analysis, the gene appears to be a composite of different syntenic fragments. For the *hemH* syntenic block (~12.7 kbp), three strains had additional genetic content between the putative esterase and putative flavin adenine dinucleotide (FAD)-linked oxidoreductase-encoding genes adjacent to *hemH*, contributing an additional 1.5 kbp (Fig. S9). Variability was also observed at both boundaries of the *hemL* syntenic block in the hi*Hh* genomes (Fig. S10). Either *hemL* or the adjacent *ata* gene, encoding the Ata adhesin autotransporter, constitutes the boundary of the syntenic block in which *hemL* resides. At the opposite boundary, variability is observed after the *manA* gene, resulting in a syntenic block that ranges in size from ~9.6 to ~14 kbp. In the hd*Hh* genomes, primarily both *ata* and *hemL* are absent from the syntenic block boundary; however, for one strain (60262_BAL_Hi3) only *hemL* was absent.

Using *de novo* assemblies, 4/7 *hem* syntenic blocks were predicted to be the same as determined using reference-assisted genome assemblies. Of the three remaining syntenic blocks (*hemE*, *hemH* and *hemL*), variation was observed in the form of a boundary shift at one end of each syntenic block, reducing their size to ~11.6, ~8.3 and ~2.8 kbp, respectively.

### Haemin biosynthesis was likely acquired from a *H. parainfluenzae* ancestor in the early stages of hi*Hh* divergent evolution

**Fig. 3.**
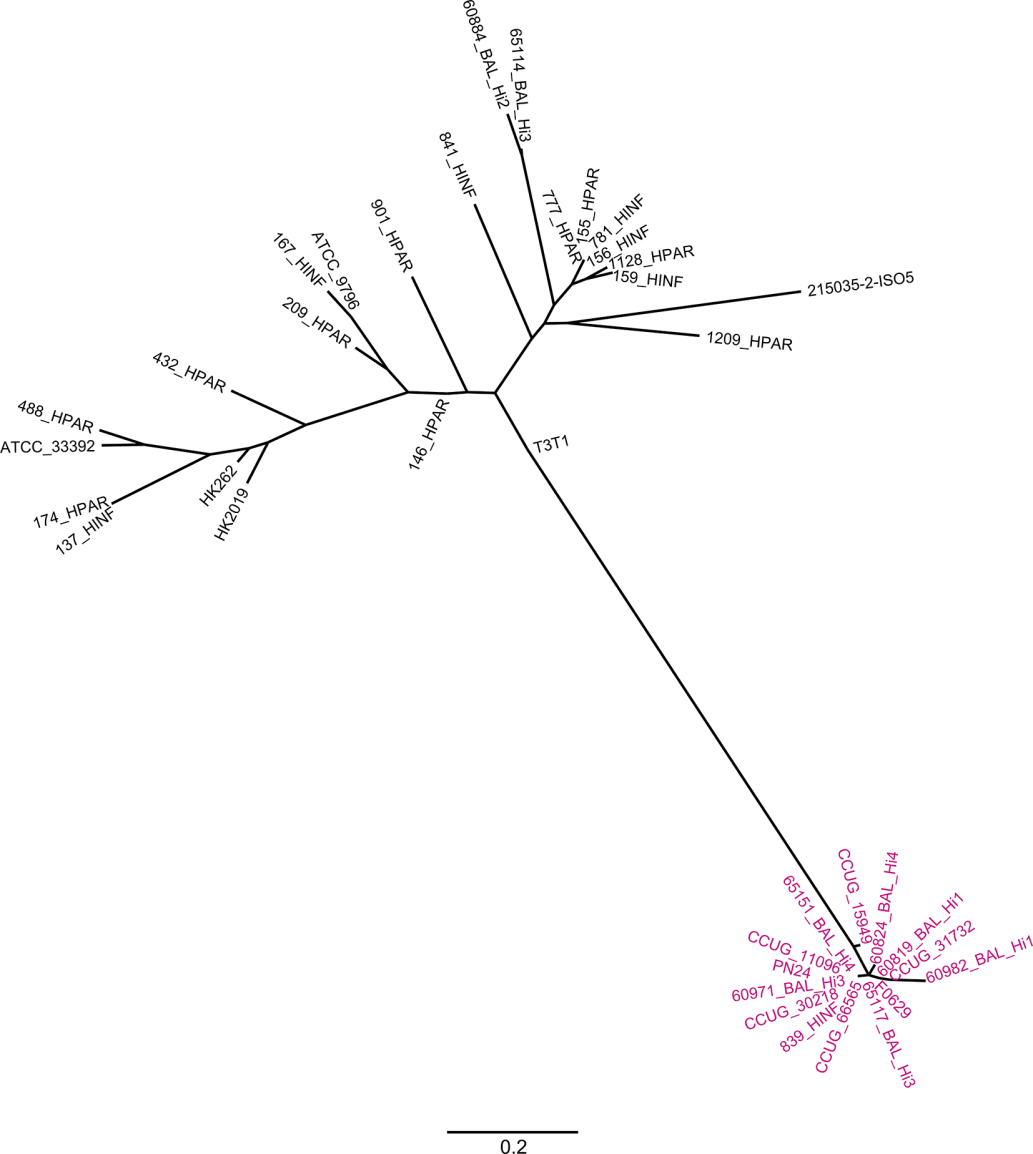
Maximum-likelihood phylogeny of haemin biosynthesis pathway genes in 14 hi*Hh* (pink) and 24 *H *. *parainfluenzae* (black), constructed using 73 orthologous, biallelic SNPs, with reference to a concatenated nucleotide sequence of haemin biosynthesis genes from *H. parainfluenzae* T3T1 (GenBank accession no. NC_015964.1). Bar, nucleotide substitutions per site.

To identify the origin of the nine *hem* genes in hi*Hh* using contemporary datasets, sequence data from hi*Hh* strain 60819_B_Hi1 were first compared to the National Center for Biotechnology Information (NCBI) nr/nt database, which contained 290 closed or draft *Haemophilus* spp. genomes (on February 2018). The *hem* gene sequences were most similar to homologues in *H. parainfluenzae* [amino acid percentage identity scores ranging between 63 % *(hemD*) to 91 % (*hemB)*], consistent with this species being most closely related to *H. haemolyticus* and *H. influenzae* at the whole-genome level (Fig. 1, Table S2). Phylogenetic analysis of the concatenated *hem* nucleotide sequences from the 14 hi*Hh* and 24 *H *. *parainfluenzae* genomes showed that all hi*Hh* isolates clustered together and were distinct from the *H. parainfluenzae* strains (Fig. 3). Taken together, these results confirm the original findings of Nørskov-Lauritsen and colleagues [3] that the hi*Hh hem* genes were not recently laterally acquired from *H. parainfluenzae *.

**Fig. 4.**
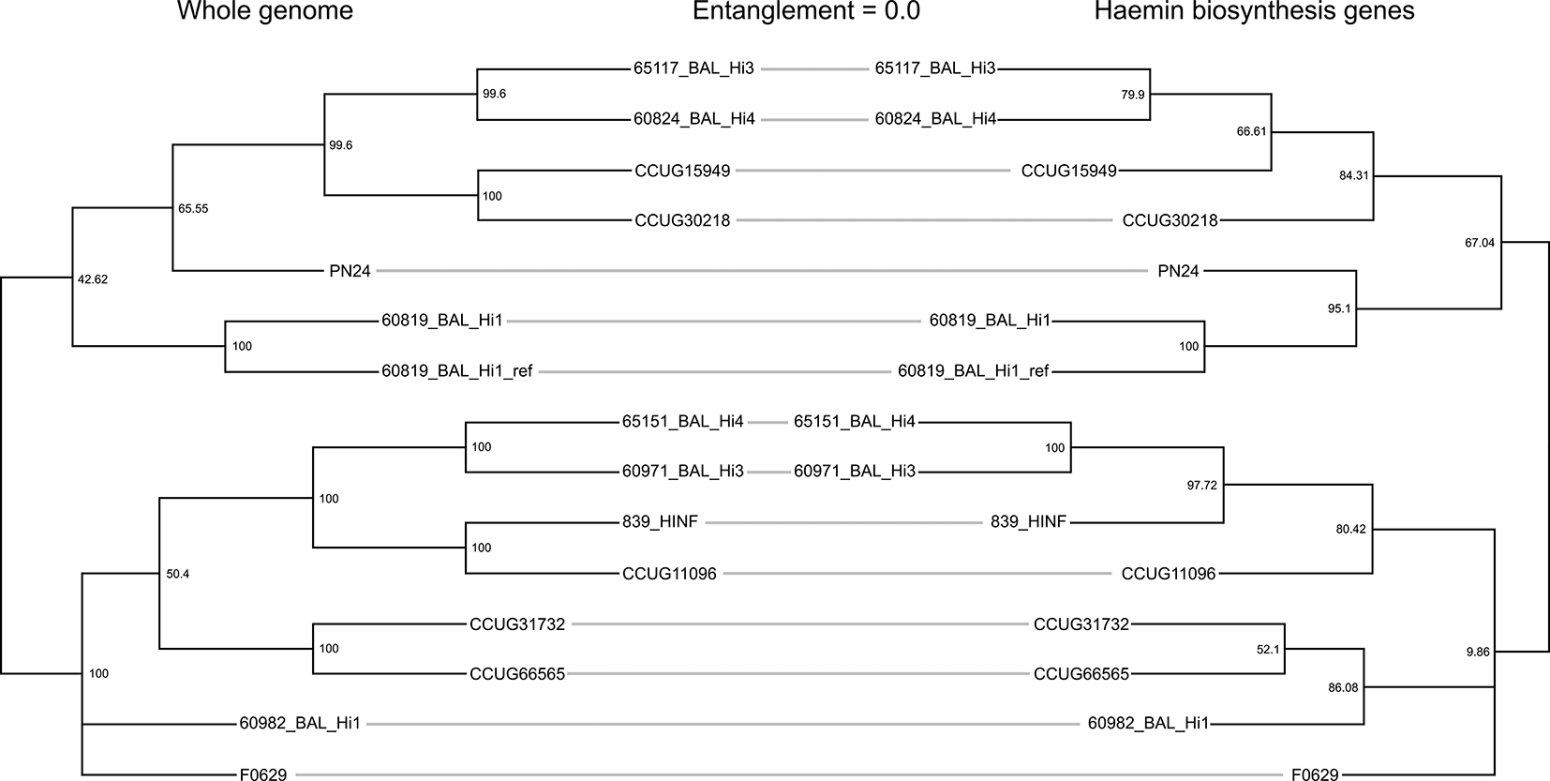
Evolution of the haemin biosynthesis pathway compared to whole-genome evolution in hi*Hh*. Midpoint-rooted maximum parsimony trees of the 14 hi*Hh* were constructed with reference to 60819_BAL_Hi1. Bootstrap values were inferred from 1000 replicates. The whole-genome phylogeny (left) was derived from 114 346 orthologous, biallelic, SNPs, using a merged, multi-contig 60819_BAL_Hi1 assembled genome as the reference. The haemin biosynthesis pathway phylogeny (right) was derived from 548 orthologous, biallelic, SNPs, with reference to a concatenated nucleotide sequence of haemin biosynthesis genes from 60819_BAL_Hi1. In the tanglegram plot, lines are used to connect the same taxa in both trees. The absence of entanglement does not reflect topological differences in the trees.

To determine whether hi*Hh hem* evolution reflected whole-genome evolution, maximum parsimony phylogenies were reconstructed using SNPs identified from both datasets and compared (Fig. 4). Whilst not identical, the phylogenies did not demonstrate any entanglement, indicating that the *hem* genes likely did not evolve independently of the rest of the genome. The minor differences in tree topologies may be explained by selective pressure to maintain haemin biosynthesis. To investigate this, dN/dS ratios were calculated for each *hem* gene in both hi*Hh* and *H. parainfluenzae* (Table S3). dN/dS scores ranged from 0.064 (*hemB*) to 0.215 (*hemG*) in hi*Hh*, and 0.025 (*hemL*) to 0.175 (*hemG*) in *H. parainfluenzae *. Housekeeping gene dN/dS ratios were comparable to those calculated for the *hem* genes in the hi*Hh* genomes, *recA*, 0.006; *adk*, 0.014; *frdB*, 0.025; *mdh*, 0.026; *pgi*, 0.051; and *atpG*, 0.692; demonstrating that the *hem* genes are under negative (purifying) selection in each of these populations, consistent with selective forces retaining the haemin biosynthesis capability in hi*Hh* and *H. parainfluenzae *.

We next investigated the similarity of gene arrangements flanking the *hem* genes in *H. parainfluenzae* and hi*Hh* to determine whether additional genetic content was shared during haemin biosynthesis acquisition in *H. haemolyticus *. Amongst the nine *hem* genes, four scenarios were observed with reference to the *H. parainfluenzae* T3T1 genome: (i) the entire syntenic block was present (*hemCD*; Fig. S3); (ii) the entire syntenic block plus additional neighbouring sequence (*hemB*, *hemN* and *hemG*; Figs S5, S8 and S7) was present; (iii) a fragment of the syntenic block was present (*hemA*, *hemE* and *hemH*; Figs S4, S6 and S9); and (iv) a fragment of the syntenic block plus additional neighbouring sequence (*hemL*; Fig. S10) was present. These observations indicate that, during haemin biosynthesis gene acquisition events, additional neighbouring coding sequences were likely also acquired, probably from the *H. parainfluenzae* ancestor.

Next, the pangenome of hi*Hh* was examined to identify potential additional instances of recombination between the *H. parainfluenzae* and hi*Hh* ancestors. Interrogation of a *H. haemolyticus* pangenome generated from 64 *H *. *haemolyticus* genomes identified 120 genes present in hi*Hh* but absent in hd*Hh*. ls-bsr comparisons of the 120 loci to the closed genome of *H. parainfluenzae* T3T1 demonstrated that 36/120 genes had a BSR ≥0.8 to orthologous coding sequences in *H. parainfluenzae *. Further pangenome interrogation identified 88 genes unique to hd*Hh*, of which 35 had a BSR ≥0.8 to orthologous coding sequences in *H. parainfluenzae *. Collectively, this demonstrates that recombination between *H. parainfluenzae* and *H. haemolyticus* has likely occurred on multiple occasions, and is not limited to the haemin biosynthesis gene cluster.

## Discussion

Haemin is required for a wide array of biological processes across all branches of life; therefore, it is not surprising that haemin biosynthesis is almost ubiquitous. For eubacteria, it is estimated that only ~13 % of species lack tetrapyrrole biosynthesis genes, the essential pathway for haemin synthesis [63]. Such organisms, including many *Haemophilus* species, have almost certainly lost the ability to synthesize haemin through evolutionary processes, most likely due to the abundance and availability of haemin in certain environmental niches, teamed with the capacity to acquire it exogenously.

In this study, we have identified an unusual *H. haemolyticus* lineage that synthesizes its own haemin, to which we have given the term hi*Hh* to most accurately reflect its phenotypic and genotypic characteristics. Forming a well-supported clade within the *H. haemolyticus* lineage, hi*Hh* taxa include isolates previously misclassified as either *H. parainfluenzae *, presumably due to colony morphology and growth factor requirements, or as the informally named ‘*H. intermedius*’ due to their high genetic similarity to *H. influenzae* and *H. haemolyticus* despite their unusual haemin independence [2]. ANI investigation of the hi*Hh* taxa confirm their *H. haemolyticus* classification, despite ANI values lower than the accepted 95 % species cut-off (range: 92.95–93.44%). The observation that hd*Hh* ANI values also straddled the recommended species cut-off indicates that a 95 % ANI cut-off is not appropriate for this species. It has previously been shown that members of *Neisseria gonorrhoeae* and *Neisseria meningitidis* can have ANI values <95 % [51], so whilst a 95 % ANI is appropriate for most bacterial species, it cannot be applied ubiquitously. Comparative genomics also confirmed that all hi*Hh* examined in this study harboured a complement of genes encoding a functional haemin biosynthesis pathway. This pathway utilizes the C_5_ branch of aminolevulinic acid synthesis and the protoporphyrin-dependent branch of protohaem synthesis in an oxygen-independent manner [63], consistent with the haemin biosynthesis pathway in the near-neighbour *H. parainfluenzae *. The presence of this suite of genes, thus, confirms the haemin-independent phenotype observed in these hi*Hh* strains.

Consistent with the lack of a complete set of *hem* genes in all *H. influenzae* and almost all *H. haemolyticus* strains characterized to date (Fig. 1), the ability to synthesize haemin was likely lost in *H. influenzae* and *H. haemolyticus* after divergence of this clade from the haemin-synthesizing *H. parainfluenzae* ancestor [1]. However, previous attempts to elucidate the genetic events associated with subsequent core *hem* gene pathway acquisition in hi*Hh* have proven elusive due to limited nucleotide sequence data, resulting in insufficient evidence of recent lateral transfer from *H. parainfluenzae* based on nucleotide comparisons [3]. Using phylogenetic approaches, including phylogenomics, our findings point towards *hem* core gene acquisition early in the divergent evolution of hi*Hh* from other *H. haemolyticus* clades, probably via lateral transfer from a *H. parainfluenzae* ancestor, rather than loss of these loci across several independent hd*Hh* clades. This hypothesis is supported by several pieces of evidence. First, a tanglegram comparing SNP phylogenies of the concatenated *hem* genes versus the whole genome (Fig. 4) demonstrated that *hem* gene diversity reflects the genomic background of the hi*Hh* strains, indicating long-term evolution of these genes in hi*Hh*. The minor topological differences in the tanglegram are likely due to fewer characters in the *hem* only dataset. Second, tBLASTn analyses of the *hem* genes across all publicly available *Haemophilus* genomes failed to identify a close genetic relative, with closest matches to *H. parainfluenzae* (range: 63 to 91 % amino acid identity), ruling out recent lateral transfer from other genome-characterized *Haemophilus* species. Third, the universal presence of the core *hem* genes in hi*Hh* strains suggests that these genes were acquired in the hi*Hh* ancestor prior to evolutionary diversification. Finally, pan-genome analysis identified 120 genes shared amongst hi*Hh* strains that were absent in hd*Hh*, with 30 % of these genes demonstrating homology to those found in the *H. parainfluenzae* T3T1 genome. Two of these genes were co-located (*scrB*) or adjacent to (*scrK*) the *hemA* syntenic block (Fig. S4), which suggests their acquisition may have occurred at the same time as the *hem* genes.

The hi*Hh hem* genes reside on seven discrete and chromosomally separated syntenic blocks, the architecture of which is principally conserved amongst hi*Hh* genomes, and reflects *hem* gene arrangement in the *H. parainfluenzae* T3T1 genome. Dispersal of *hem* genes throughout the prokaryotic chromosome is thought to be more common than arrangement as an operon [67], although the latter has been observed for a small number of bacterial species [68–70]. At face value, the distinct chromosomal locations of the *hem* genes suggest they were acquired via multiple, independent events for each syntenic block. However, taxa harbouring partial haemin biosynthesis pathways were not observed in our dataset. Transduction was also considered; however, the absence of adjacent tRNA genes suggests this acquisition mechanism is impracticable (Figs S3, S4, S5, S6, S7, S8, S9 and S10). It was hypothesized that hi*Hh* was the ancestral *H. haemolyticus* phenotype, yet the location of the hi*Hh* most recent common ancestor within the *H. haemolyticus* phylogeny indicates that *hem* genes would need to have been lost multiple times during *H. haemolyticus* evolution. However, another hypothesis is that the *hem* loci were acquired during one, or perhaps two, rare but significant recombination events between the *H. parainfluenzae* ancestor and the hi*Hh* ancestor that enabled the re-establishment of a functional haemin biosynthesis pathway in the hi*Hh* lineage. This is supported by evidence that *H. haemolyticus* can readily recombine with other *Haemophilus* spp. [71, 72] and that recombination patterns in the closely related *H. influenzae* have recently been shown to involve multiple DNA blocks across the entire chromosome rather than affecting single regions only [73]. Thus, we propose that the core *hem* genes were acquired in hi*Hh* through a recombination event with an ancestral *H. parainfluenzae* strain, with subsequent stable maintenance of the *hem* genes in this lineage.

The collection of hi*Hh* isolates examined in this study spans 37 years across four countries in three continents (Table 1), demonstrating that hi*Hh* is likely not a sporadic occurrence of a phenotypic variant. hi*Hh* are likely more abundant than previously thought, but due to phenotypic misclassification as *H. parainfluenzae* or *Haemophilus parahaemolyticus* are prone to having been inaccurately documented and, thus, under-reported. Interestingly, Australian and North American hi*Hh* isolates collected to date have all been cultured from bronchoalveolar lavage specimens, whereas the majority of the Swedish and Danish strains were cultured from infections at anatomical sites atypical for *Haemophilus* (Table 1). This differs from the standard ecological niche of hd*Hh*, where it is a commensal of the human upper respiratory tract, sharing the same ecological niche as *H. influenzae* [74].

*H. haemolyticus* is generally considered to be a commensal [74], causing disease only on rare occasions [75], with such cases often associated with underlying chronic disease [76]. Therefore, the ability to synthesize haemin is potentially advantageous for *H. haemolyticus *, enabling niche expansion into environments where haemin is limited/absent [63]. Whilst not explored in this study, the potential role of hi*Hh* in disease pathogenesis warrants exploration in order to understand its clinical relevance, and the importance of identifying hi*Hh* in a diagnostic setting. Importantly, our study confirms that comparative genomics is currently the only method for accurately identifying hi*Hh* strains, which involves detection of all nine *hem* genes in conjunction with the presence of *hypD* (* H. haemolyticus* species-specific marker) and *siaT* absence (* H. influenzae* species-specific marker) [18]. A move towards whole-genome sequencing classification of ‘fuzzy’ *Haemophilus* spp. will greatly aid in the unmasking of hi*Hh* strains across a greater spectrum of patients and geographical regions.

In summary, this study has used comparative genomics to confirm a single, unusual clade of *H. haemolyticus *, the members of which are able to synthesize their own haemin. Our study also used various comparative genomic methods to identify the evolutionary origin for the haemin biosynthesis genes in hi*Hh*, which it was not possible to elucidate using lower-resolution genotyping approaches. The ability to synthesize haemin conflicts with a key phenotype previously believed to be characteristic of *H. haemolyticus *, and provides further evidence that phenotypic tests are insufficient for accurately differentiating *Haemophilus* species. hi*Hh* is a more accurate taxonomic classification for ‘*H. intermedius*’ [2, 3], and we propose that this terminology should now be used to describe *H. haemolyticus* strains that are haemin-independent. Finally, our approach demonstrates the utility and value of comparative genomics for accurate speciation of previously described ‘fuzzy’ or informal species classifications, particularly for highly recombinogenic organisms including *Haemophilus* species, which are readily confounded by lower-resolution genotyping and phenotyping approaches.

## Data bibliography

1. Short-read sequence data for the *Haemophilus haemolyticus* strains sequenced as part of this and previous studies are available in the NCBI SRA under BioProject PRJNA509094, accession numbers are listed in Table S1 (2019).

2. The 11 haemin-independent *H. haemolyticus* draft genome assemblies generated as part of this study are available in GenBank, accession numbers are listed in Table S1 (2019).

3. Accession numbers for the publicly available *Haemophilus influenzae*, *H. haemolyticus*, *Haemophilus parainfluenzae* and *Haemophilus* spp. genomes used in this study are summarized in Table S1 (2019).

## Supplementary Data

Supplementary material 1
