# Supplementary material for: Comparative genomic analysis identifies X-factor (haemin)-independent Haemophilus haemolyticus: a formal re-classification of 'Haemophilus intermedius'
Source: Microb Genom. 2019 Dec 20;6(1):e000303. doi: 10.1099/mgen.0.000303 (PMC7067038; doi:10.1099/mgen.0.000303)
Supplement: Supplementary material 1 [file mgen-6-303-s001.pdf]

Table S1. *Haemophilus* genomes used in this study.

Strain	Source	Original species designation	Revised species designation	Alternative strain ID	Haemolysis	Accession number	Country of origin	Reference
11P18	Nasopharynx	*Haemophilus haemolyticus*	---	N/A	Unknown	NZ_LCTK01000000	USA	(25)
1P26	Nasopharynx	*Haemophilus haemolyticus*	---	N/A	Unknown	NZ_LCTI00000000	USA	(25)
27P25	Nasopharynx	*Haemophilus haemolyticus*	---	N/A	Unknown	NZ_LCTH00000000	USA	(25)
3P5	Nasopharynx	*Haemophilus haemolyticus*	---	N/A	Unknown	NZ_LCTJ00000000	USA	(25)
60041 B Hi-1	Bronchoalveolar lavage	*Haemophilus haemolyticus*	---	N/A	Negative	SRR8294020	Australia	(18)
60049 B Hi-3	Bronchoalveolar lavage	*Haemophilus haemolyticus*	---	N/A	Positive	SRR8294019	Australia	(18)
60262 B Hi-3	Bronchoalveolar lavage	*Haemophilus haemolyticus*	---	N/A	Negative	SRR2148852	Australia	(17)
60303 B Hi-2	Bronchoalveolar lavage	*Haemophilus haemolyticus*	---	N/A	Negative	SRR2148853	Australia	(17)
60303 T Hi-1	Throat	*Haemophilus haemolyticus*	---	N/A	Negative	SRR8294022	Australia	(18)
60317 T Hi-3	Throat	*Haemophilus haemolyticus*	---	N/A	Positive	SRR8294021	Australia	(18)
60370 B Hi-3	Bronchoalveolar lavage	*Haemophilus haemolyticus*	---	N/A	Positive	SRR2148854	Australia	(17)
60370 T Hi-4	Throat	*Haemophilus haemolyticus*	---	N/A	Positive	SRR8294024	Australia	(18)
60488 T Hi-1	Throat	*Haemophilus haemolyticus*	---	N/A	Positive	SRR8294023	Australia	(18)
60555 T Hi-1	Throat	*Haemophilus haemolyticus*	---	N/A	Negative	SRR8294026	Australia	(18)
60577 B Hi-4	Bronchoalveolar lavage	*Haemophilus haemolyticus*	---	N/A	Negative	SRR8294025	Australia	(18)
60578 B Hi-4	Bronchoalveolar lavage	*Haemophilus haemolyticus*	---	N/A	Negative	SRR8294018	Australia	(18)
60819 B Hi-1	Bronchoalveolar lavage	*Haemophilus haemolyticus*	*Haemophilus haemolyticus* (haemin independent)	N/A	Positive	SRR8294017, SDPA00000000	Australia	(18)
60824 B Hi-4	Bronchoalveolar lavage	*Haemophilus haemolyticus*	*Haemophilus haemolyticus* (haemin independent)	N/A	Positive	SRR8294040, SDPB00000000	Australia	(18)
60884 T Hi-2	Throat	*Haemophilus haemolyticus*	---	N/A	Positive	SRR8294039	Australia	(18)
60971 B Hi-3	Bronchoalveolar lavage	*Haemophilus haemolyticus*	*Haemophilus haemolyticus* (haemin independent)	N/A	Positive	SRR8294038, SDPC00000000	Australia	(18)
60982 B Hi-1	Bronchoalveolar lavage	*Haemophilus haemolyticus*	*Haemophilus haemolyticus* (haemin independent)	N/A	Negative	SRR8294037, SDPD00000000	Australia	(18)
61005 T Hi-1	Throat	*Haemophilus haemolyticus*	---	N/A	Negative	SRR8294044	Australia	(18)

Strain	Source	Original species designation	Revised species designation	Alternative strain ID	Haemolysis	Accession number	Country of origin	Reference
61019 B Hi-4	Bronchoalveolar lavage	*Haemophilus haemolyticus*	---	N/A	Negative	SRR8294043	Australia	(18)
61053 T Hi-1	Throat	*Haemophilus haemolyticus*	---	N/A	Negative	SRR8294042	Australia	(18)
61054 T Hi-2	Throat	*Haemophilus haemolyticus*	---	N/A	Negative	SRR8294041	Australia	(18)
61057 T Hi-5	Throat	*Haemophilus haemolyticus*	---	N/A	Negative	SRR8294046	Australia	(18)
61061 B Hi-4	Bronchoalveolar lavage	*Haemophilus haemolyticus*	---	N/A	Positive	SRR8294045	Australia	(18)
61072 B Hi-1	Bronchoalveolar lavage	*Haemophilus haemolyticus*	---	N/A	Negative	SRR8294004	Australia	(18)
61072 B Hi-4	Bronchoalveolar lavage	*Haemophilus haemolyticus*	---	N/A	Negative	SRR8294005	Australia	(18)
61077 B Hi-1	Bronchoalveolar lavage	*Haemophilus haemolyticus*	---	N/A	Negative	SRR8294002	Australia	(18)
65117 B Hi-3	Bronchoalveolar lavage	*Haemophilus haemolyticus*	*Haemophilus haemolyticus* (haemin independent)	N/A	Negative	SRR8294003, SDPE00000000	Australia	(18)
65123 T Hi-3	Throat	*Haemophilus haemolyticus*	---	N/A	Positive	SRR8294008	Australia	(18)
65141 B Hi-3	Bronchoalveolar lavage	*Haemophilus haemolyticus*	---	N/A	Positive	SRR8294009	Australia	(18)
65151 B Hi-4	Bronchoalveolar lavage	*Haemophilus haemolyticus*	*Haemophilus haemolyticus* (haemin independent)	N/A	Negative	SRR8294006, SDPF00000000	Australia	(18)
839_HINF*	Bronchoalveolar lavage	*Haemophilus influenzae*	*Haemophilus haemolyticus* (haemin independent)	N/A		NZ_JURC00000000	USA	(24)
88002 N Hi-1	Nasopharynx	*Haemophilus haemolyticus*	---	N/A	ND	SRR8294007	Australia	(18)
90691 N Hi-1	Nasopharynx	*Haemophilus haemolyticus*	---	N/A	Negative	SRR8294000	Australia	(18)
ATCC 33390	Sputum	*Haemophilus haemolyticus*	---	Hh33390; NCTC 10659	Negative	SRR8294001	UK	(18)
C1*	Bronchoalveolar lavage	*Haemophilus* sp.	*Haemophilus haemolyticus*	N/A		NZ_LDVZ00000000	Australia	(23)
CCUG 11096*	Pleural fluid	*Haemophilus intermedius* subsp. *intermedius*	*Haemophilus haemolyticus* (haemin independent)	N/A		SRR8294030, SDPG00000000	Sweden	(3)
CCUG 15949*	Eye	*Haemophilus intermedius* subsp. *intermedius*	*Haemophilus haemolyticus* (haemin independent)	N/A		SRR8294029, SDPH00000000	Sweden	(3)
CCUG 30218*	Cerebrospinal fluid	*Haemophilus intermedius* subsp. *intermedius*	*Haemophilus haemolyticus* (haemin independent)	N/A		SRR8294032, SDPI00000000	Sweden	(3)
CCUG 31732*	Ascitic fluid	*Haemophilus intermedius* subsp. *intermedius*	*Haemophilus haemolyticus* (haemin independent)	N/A		SRR8294031, SDPJ00000000	Sweden	(3)

Strain	Source	Original species designation	Revised species designation	Alternative strain ID	Haemolysis	Accession number	Country of origin	Reference
CCUG 66565*	---	*Haemophilus* sp.	*Haemophilus haemolyticus* (haemin independent)	N/A		LZOX00000000	Sweden	Unpublished
F0397*	---	*Haemophilus* sp.	*Haemophilus haemolyticus*	oral taxon 851 strain F0397	Unknown	AGRK01000000	USA	Unpublished
F0629*	---	*Haemophilus* sp.	*Haemophilus haemolyticus* (haemin independent)	oral taxon 036 strain F0629	Unknown	NZ_CP027235.1	USA	Unpublished
H12	Nasopharynx	*Haemophilus haemolyticus*	---	N/A	Negative	SRR8294034	Australia	(18)
H187	Nasopharynx	*Haemophilus haemolyticus*	---	N/A	Negative	SRR8294033	Australia	(18)
H19	Nasopharynx	*Haemophilus haemolyticus*	---	N/A	Negative	SRR8294036	Australia	(18)
H274	Nasopharynx	*Haemophilus haemolyticus*	---	N/A	Positive	SRR8294035	Australia	(18)
H42	Nasopharynx	*Haemophilus haemolyticus*	---	N/A	Negative	SRR8294028	Australia	(18)
H51	Nasopharynx	*Haemophilus haemolyticus*	---	N/A	Negative	SRR8294027	Australia	(18)
H54	Nasopharynx	*Haemophilus haemolyticus*	---	N/A	Negative	SRR8294010	Australia	(18)
H56	Nasopharynx	*Haemophilus haemolyticus*	---	N/A	Negative	SRR8294011	Australia	(18)
H6	Nasopharynx	*Haemophilus haemolyticus*	---	N/A	Positive	SRR8294012	Australia	(18)
H9	Nasopharynx	*Haemophilus haemolyticus*	---	N/A	Negative	SRR8294013	Australia	(18)
HI2028	Blood	*Haemophilus haemolyticus*	---	N/A	Negative	SRR8294014	USA	(18)
Hi-S080	Blood	*Haemophilus haemolyticus*	---	N/A	Unknown	SRR8294015	Australia	(18)
M19107	Oropharynx	*Haemophilus haemolyticus*	---	N/A	Unknown	NZ_AFQN00000000	USA	(22)
M19501	Oropharynx	*Haemophilus haemolyticus*	---	N/A	Unknown	NZ_AFQN00000000	USA	(22)
M21127	Blood	*Haemophilus haemolyticus*	---	N/A	Unknown	NZ_AFQP00000000	USA	(22)
M21621	Knee fluid	*Haemophilus haemolyticus*	---	N/A	Unknown	NZ_AFQQ00000000	USA	(22)
M21639	Blood	*Haemophilus haemolyticus*	---	N/A	Unknown	NZ_AFQR00000000	USA	(22)
PN24*	Urine	*Haemophilus intermedius* subsp. *intermedius*	*Haemophilus haemolyticus* (haemin independent)	N/A		SRR8294016, SDPK00000000	Denmark	(3)
1128_HPAR	Bronchial brush	*Haemophilus parainfluenzae*	---	N/A		JWCE00000000	USA	(24)
1209_HPAR	Bronchoalveolar lavage	*Haemophilus parainfluenzae*	---	N/A		JVYT00000000	USA	(24)
137_HINF*	Bronchoalveolar lavage	*Haemophilus influenzae*	*Haemophilus parainfluenzae*	N/A		JVSU00000000	USA	(24)
146_HPAR	Bronchoalveolar lavage	*Haemophilus parainfluenzae*	---	N/A		JVSL00000000	USA	(24)

Strain	Source	Original species designation	Revised species designation	Alternative strain ID	Haemolysis	Accession number	Country of origin	Reference
155_HPAR	Bronchoalveolar lavage	*Haemophilus parainfluenzae*	---	N/A		JVSB00000000	USA	(24)
156_HINF*	---	*Haemophilus influenzae*	*Haemophilus parainfluenzae*	N/A		JVSA00000000	USA	(24)
159_HINF*	---	*Haemophilus influenzae*	*Haemophilus parainfluenzae*	N/A		JVRW00000000	USA	(24)
167_HINF*	Wound	*Haemophilus influenzae*	*Haemophilus parainfluenzae*	N/A		JVRO00000000	USA	(24)
174_HPAR	Bronchoalveolar lavage	*Haemophilus parainfluenzae*	---	N/A		JVRH00000000	USA	(24)
209_HPAR	Bronchoalveolar lavage	*Haemophilus parainfluenzae*	---	N/A		JVPX00000000	USA	(24)
215035-2-ISO5	---	*Haemophilus parainfluenzae*	---	N/A		LFXN01000000	USA	Unpublished
432_HPAR	Bronchoalveolar lavage	*Haemophilus parainfluenzae*	---	N/A		JVHG00000000	USA	(24)
488_HPAR	Bronchoalveolar lavage	*Haemophilus parainfluenzae*	---	N/A		JVFE00000000	USA	(24)
60884 B Hi-2	Bronchoalveolar lavage	*Haemophilus parainfluenzae*	---	N/A		MPJJ00000000	Australia	(18)
65114 B Hi-3	Bronchoalveolar lavage	*Haemophilus parainfluenzae*	---	N/A		MPJK00000000	Australia	(18)
777_HPAR	Bronchoalveolar lavage	*Haemophilus parainfluenzae*	---	N/A		JUTJ00000000	USA	(24)
781_HINF*	Bronchoalveolar lavage	*Haemophilus influenzae*	*Haemophilus parainfluenzae*	N/A		JUTE00000000	USA	(24)
841_HINF*	Bronchoalveolar lavage	*Haemophilus influenzae*	*Haemophilus parainfluenzae*	N/A		JUQZ00000000	USA	(24)
901_HPAR	Bronchoalveolar lavage	*Haemophilus parainfluenzae*	---	N/A		JUOR00000000	USA	(24)
ATCC 9796	---	*Haemophilus parainfluenzae*	---	655		MAQD00000000	Unknown	(18)
ATCC 33392	---	*Haemophilus parainfluenzae*	---	NCTC 7857; DSM 8978; CIP 102513; CCUG 12836; HK409		AEWU01000000	Unknown	Unpublished
HK2019	---	*Haemophilus parainfluenzae*	---	N/A		AJTC01000000	Uknown	Unpublished
HK262	---	*Haemophilus parainfluenzae*	---	N/A		AJMW01000000	Unknown	Unpublished
T3T1	---	*Haemophilus parainfluenzae*	---	N/A		NC_015964.1	UK	Unpublished
3655	Otitis media	*Haemophilus influenzae*	---	CGSHi3655		NZ_AAZF00000000	USA	(27)
10810	---	*Haemophilus influenzae*	---	N/A		NC_016809	Undetermined	Unpublished
1057_HINF	Sputum	*Haemophilus influenzae*	---	N/A		NZ_JWEN00000000	USA	(24)
1059_HINF	Sputum	*Haemophilus influenzae*	---	N/A		NZ_JWEL00000000	USA	(24)
1061_HINF	---	*Haemophilus influenzae*	---	N/A		NZ_JWEJ00000000	USA	(24)
1123_HINF	Bronchoalveolar lavage	*Haemophilus influenzae*	---	N/A		NZ_JWCI00000000	USA	(24)
1124_HINF	Bronchoalveolar lavage	*Haemophilus influenzae*	---	N/A		NZ_JWCH00000000	USA	(24)
177_HINF	Sputum	*Haemophilus influenzae*	---	N/A		NZ_JVRF00000000	USA	(24)

Strain	Source	Original species designation	Revised species designation	Alternative strain ID	Haemolysis	Accession number	Country of origin	Reference
22.1-21	Nasopharynx	*Haemophilus influenzae*	---	CGSHi22121		AAZD00000000	USA	(27)
22.4-21	Nasopharynx	*Haemophilus influenzae*	---	CGSHi22421		NZ_AAZJ00000000	USA	(27)
40_HINF	Sputum	*Haemophilus influenzae*	---	N/A		NZ_JVIR00000000	USA	(24)
492_HINF	Sputum	*Haemophilus influenzae*	---	N/A		NZ_JVEZ00000000	USA	(24)
536_HINF	Bronchoalveolar lavage	*Haemophilus influenzae*	---	N/A		NZ_JVDE00000000	USA	(24)
552_HINF	Bronchoalveolar lavage	*Haemophilus influenzae*	---	N/A		NZ_JVCL00000000	USA	(24)
614_HPAR*	Sputum	*Haemophilus parainfluenzae*	*Haemophilus influenzae*	N/A		NZ_JUZZ00000000	USA	(24)
615_HINF	Sputum	*Haemophilus influenzae*	---	N/A		NZ_JUZY00000000	USA	(24)
6P18H1	Sputum	*Haemophilus influenzae*	---	N/A		NZ_ABWW00000000	USA	(26)
7P49H1	Sputum	*Haemophilus influenzae*	---	N/A		NZ_ABWV00000000	USA	(26)
86-028NP	Nasopharynx	*Haemophilus influenzae*	---	N/A		NC_007146	USA	(28)
ATCC 11116	---	*Haemophilus influenzae*	---	CCUG 25716; NCTC 8502		NZ_AFBC01000000	USA	Unpublished
ATCC 8142	---	*Haemophilus influenzae*	---	Gifu199; GTC_15022		DRX014253	USA	Unpublished
ATCC 9007	---	*Haemophilus influenzae*	---	Gifu101; GTC_15020		DRX014159	USA	Unpublished
ATCC 9327	---	*Haemophilus influenzae*	---	Gifu098; GTC_15021		DRX014438	USA	Unpublished
ATCC 9332	---	*Haemophilus influenzae*	---	Gifu102; GTC_15019		DRX014160	Undetermined	Unpublished
ATCC 9795	---	*Haemophilus influenzae*	---	Gifu100; GTC_15028		DRX014158	USA	Unpublished
ATCC 9833	---	*Haemophilus influenzae*	---	Gifu103; GTC_15017		DRX014161	USA	Unpublished
C10	Bronchoalveolar lavage	*Haemophilus influenzae*	---	N/A		NZ_LDWA00000000	Australia	(23)
C486	---	*Haemophilus influenzae*	---	N/A		NZ_CP007471	USA	Unpublished
CGSHiCZ412602	---	*Haemophilus influenzae*	---	N/A		CP007805	Czech Republic	Unpublished
DC7331102	Invasive disease	*Haemophilus influenzae*	---	N/A		ERR125109	UK	(36)
F3031	Brazilian purpuric fever	*Haemophilus influenzae*	---	N/A		NC_014920	Brazil	(29)
F3047	Conjunctivitis	*Haemophilus influenzae*	---	N/A		NC_014922	Brazil	(29)
GTC 15012	---	*Haemophilus influenzae*	---	Gifu099		DRX014157	Undetermined	Unpublished
H18	---	*Haemophilus influenzae*	---	N/A		Unpublished	Australia	Unpublished
H180	---	*Haemophilus influenzae*	---	N/A		Unpublished	Australia	Unpublished
H40	---	*Haemophilus influenzae*	---	N/A		Unpublished	Australia	Unpublished

Strain	Source	Original species designation	Revised species designation	Alternative strain ID	Haemolysis	Accession number	Country of origin	Reference
Hi1008	Otitis media	*Haemophilus influenzae*	---	N/A		ERR125030	Finland	(36)
Hi11	Carriage	*Haemophilus influenzae*	---	N/A		ERR125052	Finland	(36)
Hi1104	Sputum	*Haemophilus influenzae*	---	N/A		NZ_JFZM00000000	Spain	(30)
Hi1124	Otitis media	*Haemophilus influenzae*	---	N/A		ERR125031	Finland	(36)
Hi1158	Otitis media	*Haemophilus influenzae*	---	N/A		ERR125032	Finland	(36)
Hi1180	Otitis media	*Haemophilus influenzae*	---	N/A		ERR125033	Finland	(36)
Hi1200	Otitis media	*Haemophilus influenzae*	---	N/A		ERR125034	Finland	(36)
Hi1207	Otitis media	*Haemophilus influenzae*	---	N/A		ERR125035	Finland	(36)
Hi1209	---	*Haemophilus influenzae*	---	N/A		JMQP01000000	Finland	Unpublished
Hi1231	Otitis media	*Haemophilus influenzae*	---	N/A		ERR125036	Finland	(36)
Hi1233	Otitis media	*Haemophilus influenzae*	---	N/A		ERR125037	Finland	(36)
Hi1247	Otitis media	*Haemophilus influenzae*	---	N/A		ERR125038	Finland	(36)
Hi1268	Otitis media	*Haemophilus influenzae*	---	N/A		ERR125039	Finland	(36)
Hi1363	Otitis media	*Haemophilus influenzae*	---	N/A		ERR125043	Finland	(36)
HI1388	Ear	*Haemophilus influenzae*	---	3A		LFDN00000000	USA	(31)
HI1413	Ear	*Haemophilus influenzae*	---	N/A		LHSM00000000	USA	(31)
HI1426	Cerebrospinal fluid	*Haemophilus influenzae*	---	1396B		LFDL00000000	USA	(31)
Hi1500	Chronic obstructive pumonary disease	*Haemophilus influenzae*	---	N/A		ERR125069	Spain	(36)
Hi1513	Chronic obstructive pumonary disease	*Haemophilus influenzae*	---	N/A		ERR125070	Spain	(36)
Hi1549	Chronic obstructive pumonary disease	*Haemophilus influenzae*	---	N/A		ERR125071	Spain	(36)
Hi1553	Chronic obstructive pumonary disease	*Haemophilus influenzae*	---	N/A		ERR125072	Spain	(36)
Hi1556	Chronic obstructive pumonary disease	*Haemophilus influenzae*	---	N/A		ERR125073	Spain	(36)
Hi1557	Chronic obstructive pumonary disease	*Haemophilus influenzae*	---	N/A		ERR125074	Spain	(36)
Hi1558	Chronic obstructive pumonary disease	*Haemophilus influenzae*	---	N/A		ERR125075	Spain	(36)
Hi1559	Chronic obstructive pumonary disease	*Haemophilus influenzae*	---	N/A		ERR125076	Spain	(36)
Hi1560	Chronic obstructive pumonary disease	*Haemophilus influenzae*	---	N/A		ERR125077	Spain	(36)
Hi1566	Chronic obstructive pumonary disease	*Haemophilus influenzae*	---	N/A		ERR125078	Spain	(36)
Hi1568	Chronic obstructive pumonary disease	*Haemophilus influenzae*	---	N/A		ERR125079	Spain	(36)

Strain	Source	Original species designation	Revised species designation	Alternative strain ID	Haemolysis	Accession number	Country of origin	Reference
Hi16	Carriage	*Haemophilus influenzae*	---	N/A		ERR125053	Finland	(36)
Hi1606	Chronic obstructive pulmonary disease	*Haemophilus influenzae*	---	N/A		ERR125080	Spain	(36)
Hi1607	Chronic obstructive pulmonary disease	*Haemophilus influenzae*	---	N/A		ERR125081	Spain	(36)
Hi1619	Chronic obstructive pulmonary disease	*Haemophilus influenzae*	---	N/A		ERR125082	Spain	(36)
Hi162	Otitis media	*Haemophilus influenzae*	---	N/A		ERR125020	Finland	(36)
Hi1621	Chronic obstructive pulmonary disease	*Haemophilus influenzae*	---	N/A		ERR125083	Spain	(36)
Hi1622	Chronic obstructive pulmonary disease	*Haemophilus influenzae*	---	N/A		ERR125084	Spain	(36)
Hi1623	Chronic obstructive pulmonary disease	*Haemophilus influenzae*	---	N/A		ERR125085	Spain	(36)
Hi1630	Chronic obstructive pulmonary disease	*Haemophilus influenzae*	---	N/A		ERR125086	Spain	(36)
Hi167	Otitis media	*Haemophilus influenzae*	---	N/A		ERR125050	Finland	(36)
Hi17	Carriage	*Haemophilus influenzae*	---	N/A		ERR125054	Finland	(36)
Hi176	Otitis media	*Haemophilus influenzae*	---	N/A		ERR125021	Finland	(36)
Hi177	Carriage	*Haemophilus influenzae*	---	N/A		ERR125057	Finland	(36)
HI1980	Blood	*Haemophilus influenzae*	---	N/A		NZ_LFFO00000000	USA	(32)
Hi199	Otitis media	*Haemophilus influenzae*	---	N/A		ERR125049	Finland	(36)
Hi2019	Otitis media	*Haemophilus influenzae*	---	N/A		ERR125041	Finland	(36)
Hi206	Otitis media	*Haemophilus influenzae*	---	N/A		ERR125045	Finland	(36)
HI2192	---	*Haemophilus influenzae*	---	N/A		NZ_LHSN00000000	USA	Unpublished
Hi24	Carriage	*Haemophilus influenzae*	---	N/A		ERR125055	Finland	(36)
HI2428	---	*Haemophilus influenzae*	---	N/A		NZ_LHSO00000000	USA	Unpublished
Hi264	Carriage	*Haemophilus influenzae*	---	N/A		ERR125058	Finland	(36)
Hi285	Otitis media	*Haemophilus influenzae*	---	N/A		ERR125022	Finland	(36)
Hi322	Cerebrospinal fluid	*Haemophilus influenzae*	---	N/A		NZ_JXLX00000000	Italy	(33)
Hi345	Blood	*Haemophilus influenzae*	---	N/A		NZ_JXLY00000000	Italy	(33)
Hi359	Blood	*Haemophilus influenzae*	---	N/A		NZ_JXLZ00000000	Italy	(33)
Hi361	Blood	*Haemophilus influenzae*	---	N/A		NZ_JXMA00000000	Italy	(33)
Hi375	Otitis media	*Haemophilus influenzae*	---	N/A		ERR125023	Finland	(36)
Hi378	Cerebrospinal fluid	*Haemophilus influenzae*	---	N/A		NZ_JXMB00000000	Italy	(33)

Strain	Source	Original species designation	Revised species designation	Alternative strain ID	Haemolysis	Accession number	Country of origin	Reference
Hi381	Cerebrospinal fluid	*Haemophilus influenzae*	---	N/A		NZ_JXMC00000000	Italy	(33)
Hi394	Blood	*Haemophilus influenzae*	---	N/A		NZ_JXMD00000000	Italy	(33)
Hi403	Cerebrospinal fluid	*Haemophilus influenzae*	---	N/A		NZ_JXME00000000	Italy	(33)
Hi411	Sputum	*Haemophilus influenzae*	---	N/A		NZ_JFZK00000000	Spain	(30)
Hi432	Otitis media	*Haemophilus influenzae*	---	N/A		ERR125024	Finland	(36)
Hi443	Carriage	*Haemophilus influenzae*	---	N/A		ERR125059	Finland	(36)
Hi477	Otitis media	*Haemophilus influenzae*	---	N/A		ERR125025	Finland	(36)
Hi486	Otitis media	*Haemophilus influenzae*	---	N/A		ERR125026	Finland	(36)
Hi492	Carriage	*Haemophilus influenzae*	---	N/A		ERR125060	Finland	(36)
Hi525	Carriage	*Haemophilus influenzae*	---	N/A		ERR125061	Finland	(36)
Hi584	Sputum	*Haemophilus influenzae*	---	N/A		NZ_JFZL00000000	Spain	(30)
Hi609	Carriage	*Haemophilus influenzae*	---	N/A		ERR125062	Finland	(36)
Hi639	Otitis media	*Haemophilus influenzae*	---	N/A		ERR125044	Finland	(36)
Hi658	Otitis media	*Haemophilus influenzae*	---	N/A		ERR125046	Finland	(36)
Hi667	Otitis media	*Haemophilus influenzae*	---	N/A		ERR125027	Finland	(36)
Hi709	Carriage	*Haemophilus influenzae*	---	N/A		ERR125063	Finland	(36)
Hi723	Otitis media	*Haemophilus influenzae*	---	N/A		ERR125028	Finland	(36)
Hi740	Otitis media	*Haemophilus influenzae*	---	N/A		ERR125048	Finland	(36)
Hi787	Carriage	*Haemophilus influenzae*	---	N/A		ERR125064	Finland	(36)
Hi794	Carriage	*Haemophilus influenzae*	---	N/A		ERR125065	Finland	(36)
Hi805	Carriage	*Haemophilus influenzae*	---	N/A		ERR125066	Finland	(36)
Hi88	Carriage	*Haemophilus influenzae*	---	N/A		ERR125056	Finland	(36)
Hi968	Chronic obstructive pulmonary disease	*Haemophilus influenzae*	---	N/A		ERR125067	Spain	(36)
Hi973	Otitis media	*Haemophilus influenzae*	---	N/A		ERR125047	Finland	(36)
Hi981	Otitis media	*Haemophilus influenzae*	---	N/A		ERR125029	Finland	(36)
KR494	Necrotising Myositis	*Haemophilus influenzae*	---	N/A		NC_022356	Sweden	(34)
MiHi270	Nasopharynx	*Haemophilus influenzae*	---	N/A		NZ_JXMF00000000	Italy	(33)
MiHi64	Nasopharynx	*Haemophilus influenzae*	---	N/A		NZ_JXMG00000000	Italy	(33)

Strain	Source	Original species designation	Revised species designation	Alternative strain ID	Haemolysis	Accession number	Country of origin	Reference
NCTC 8143	---	*Haemophilus influenzae*	---	ATCC 33391; DSM 4690; 680		LN831035	UK	Unpublished
NT127	---	*Haemophilus influenzae*	---	N/A		NZ_ACSL00000000	USA	Unpublished
PittAA	Otitis media	*Haemophilus influenzae*	---	CGSHiAA		NZ_AAZG00000000	USA	(27)
PittEE	Otitis media	*Haemophilus influenzae*	---	CGSHiEE		NC_009566	USA	(27)
PittHH	Otitis media	*Haemophilus influenzae*	---	CGSHiHH		NZ_AAZH00000000	USA	(27)
PittII	Otorrhea	*Haemophilus influenzae*	---	CGSHiII		NZ_AAZI00000000	USA	(27)
R2846	---	*Haemophilus influenzae*	---	N/A		NC_017452	USA	Unpublished
R2866	---	*Haemophilus influenzae*	---	N/A		NC_017451	USA	Unpublished
Rd KW20	---	*Haemophilus influenzae*	---	N/A		NC_000907	Undetermined	(35)
RdAW	---	*Haemophilus influenzae*	---	N/A		NZ_ACSM00000000	Undetermined	Unpublished
RM600672	Other	*Haemophilus influenzae*	---	N/A		ERR125088	Undetermined	(36)
RM601173	Invasive	*Haemophilus influenzae*	---	N/A		ERR125089	UK	(36)
RM601974	Invasive	*Haemophilus influenzae*	---	N/A		ERR125090	UK	(36)
RM603375	Other	*Haemophilus influenzae*	---	N/A		ERR125091	UK	(36)
RM605177	Invasive	*Haemophilus influenzae*	---	N/A		ERR125092	UK	(36)
RM701878	Invasive	*Haemophilus influenzae*	---	N/A		ERR125110	Ghana	(36)
RM702879	Invasive	*Haemophilus influenzae*	---	N/A		ERR125093	PNG	(36)
RM702980	Invasive	*Haemophilus influenzae*	---	N/A		ERR125094	PNG	(36)
RM706883	Other	*Haemophilus influenzae*	---	N/A		ERR125096	PNG	(36)
RM712284	Invasive	*Haemophilus influenzae*	---	N/A		ERR125097	Australia	(36)
RM730885	Carriage	*Haemophilus influenzae*	---	N/A		ERR125098	South Korea	(36)
RM730986	Carriage	*Haemophilus influenzae*	---	N/A		ERR125099	South Korea	(36)
RM734787	Other	*Haemophilus influenzae*	---	N/A		ERR125100	USA	(36)
RM744888	Invasive	*Haemophilus influenzae*	---	N/A		ERR125101	Iceland	(36)
RM745989	Invasive	*Haemophilus influenzae*	---	N/A		ERR125102	Iceland	(36)
RM746590	Invasive	*Haemophilus influenzae*	---	N/A		ERR125103	Iceland	(36)
RM747791	Other	*Haemophilus influenzae*	---	N/A		ERR125104	Iceland	(36)
RM749092	Invasive	*Haemophilus influenzae*	---	N/A		ERR125105	RSA	(36)

Strain	Source	Original species designation	Revised species designation	Alternative strain ID	Haemolysis	Accession number	Country of origin	Reference
RM761793	Invasive	*Haemophilus influenzae*	---	N/A		ERR125106	Malaysia	(36)
RM763794	Other	*Haemophilus influenzae*	---	N/A		ERR125107	China	(36)
RM787695	Other	*Haemophilus influenzae*	---	N/A		ERR125108	Undetermined	(36)
RMHi93	Oropharynx	*Haemophilus influenzae*	---	N/A		NZ_JXMH00000000	Italy	(33)

* Denotes incorrect or incomplete species designations in prior studies

Table S2. tBLASTn percentage identity scores of hem genes from the *Haemophilus parainfluenzae* T3T1 genome compared to the haemin-independent *Haemophilus haemolyticus* (hi*Hh*) genomes.

Isolate	*hemA*	*hemL*	*hemB*	*hemC*	*hemD*	*hemE*	*hemN*	*hemG*	*hemH*
60819_B_Hi1	82.19	88.46	91.76	84.47	63.67	90.4	82.86	68.86	80.5
60824_B_Hi4	81.74	88.46	91.47	85.11	63.67	90.4	83.3	71.86	80.19
60971_B_Hi3	81.96	88.22	91.47	84.47	63.67	90.11	83.74	70.66	79.25
60982_B_Hi1	81.74	87.98	91.47	84.79	63.27	90.68	83.08	70.66	80.5
65117_B_Hi3	81.96	88.46	91.47	84.79	63.67	89.83	83.3	70.66	79.87
65151_B_Hi4	81.96	88.22	91.47	84.47	63.67	90.11	83.74	70.66	79.25
839_HINF	82.19	88.22	91.76	84.47	63.27	90.4	82.86	70.66	79.87
CCUG_11096	81.51	88.22	91.76	84.79	64.08	90.4	82.86	70.66	80.19
CCUG_15949	82.19	88.22	91.47	84.79	63.67	89.83	83.3	70.66	79.25
CCUG_30218	82.19	88.46	91.76	84.79	63.67	90.11	83.08	70.66	79.87
CCUG_31732	81.96	88.46	91.47	85.11	63.27	90.4	82.64	69.46	79.87
CCUG_66565	81.96	88.46	91.47	84.79	63.27	90.4	82.86	69.46	80.19
F0629	82.19	88.46	91.18	85.11	64.08	90.11	83.08	70.66	78.18
PN24	81.96	88.46	91.76	84.14	63.67	90.4	82.42	70.06	79.19

Table S3. dN/dS values of *hem* genes.

Gene	hi*Hh*[a]	*H. parainfluenzae*[b]
hemA	0.098	0.060
hemL	0.120	0.025
hemB	0.064	0.031
hemC	0.181	0.055
hemD	0.205	0.125
hemE	0.097	0.030
hemN	0.103	0.041
hemG	0.215	0.175
hemH	0.160	0.079

[a] Values determined using gene sequences from the 14 haemin-independent *H. haemolyticus* (hi*Hh*) genomes in this study (Table S1).

[b] Values determined using gene sequences from the 24 *H. parainfluenzae* genomes in this study (Table S1).

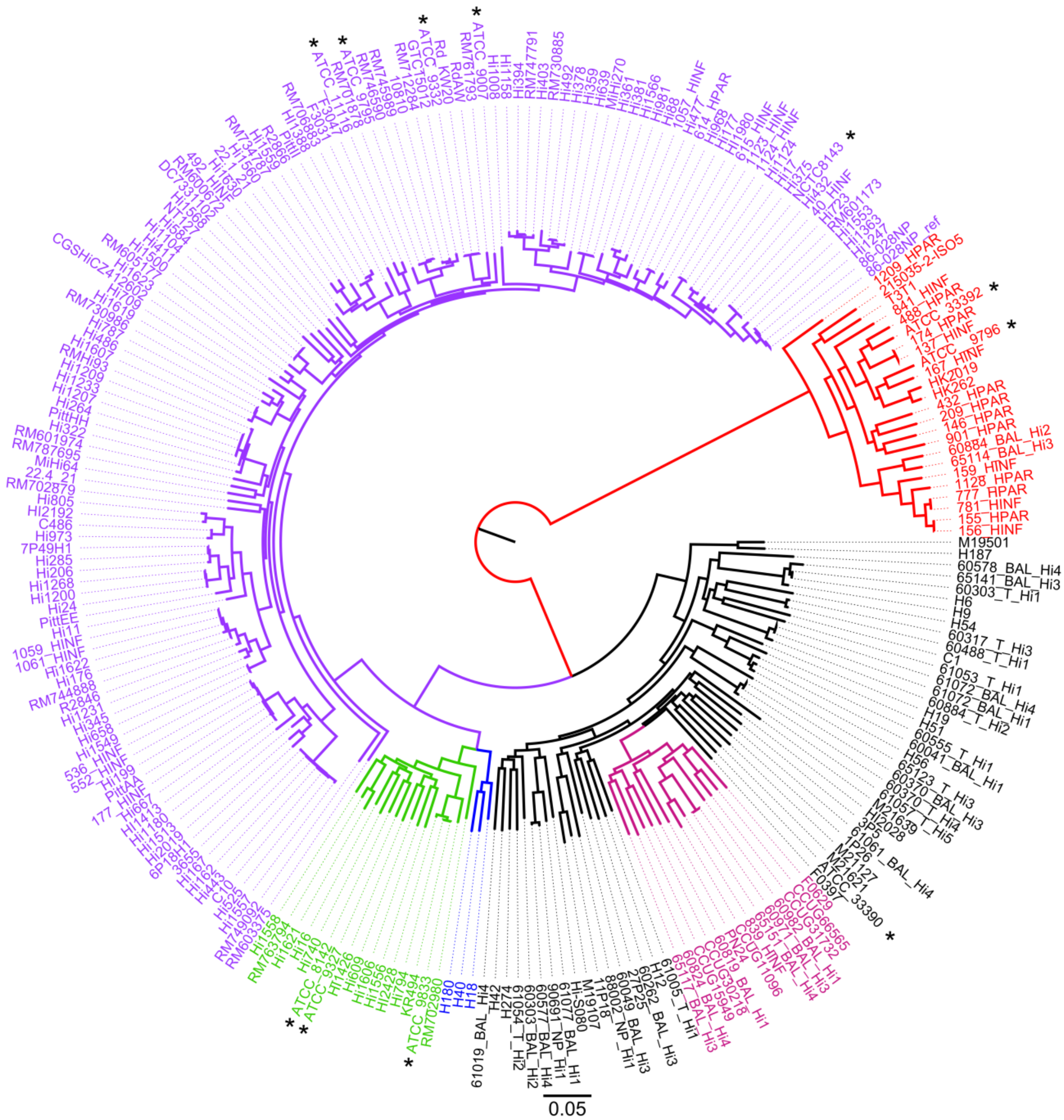

Figure S1. Maximum likelihood phylogenomic analysis verifies maximum parsimony phylogenomy. A midpoint-rooted maximum likelihood tree was constructed using 30,345 orthologous biallelic single nucleotide polymorphisms found among 152 *H. influenzae* (purple), including 16 clade I (green) (36) and three *fucP*-negative clade (blue) (17, 18) genomes, 64 *H. haemolyticus* (black) including 14 hi*Hh* and "*H. intermedius*" genomes (pink), and 24 *H. parainfluenzae* genomes (red). Type strains are denoted with an asterisk (*). The scale bar indicates 0.05 substitutions per site.

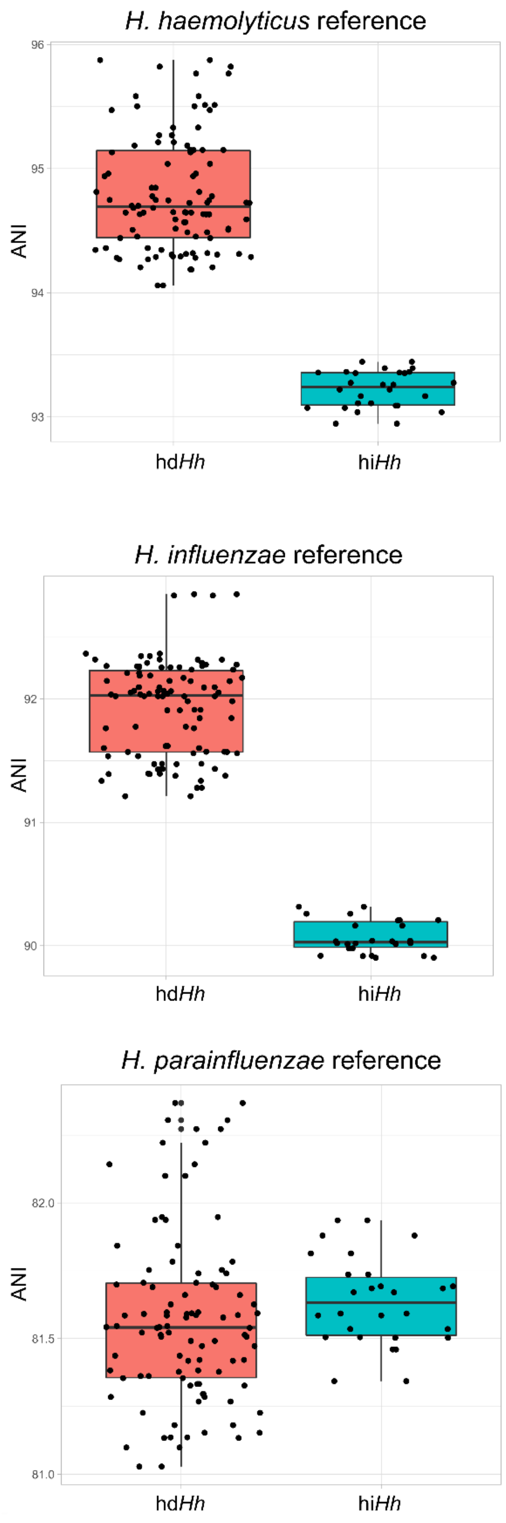

Figure S2. Average nucleotide identity values for the 64 *H. haemolyticus* clade genomes with reference to the NCTC 10839 *H. haemolyticus*, 86-028NP non-typeable *H. influenzae*, and T3T1 *H. parainfluenzae* genomes. hd*Hh*, haemin-dependent *H. haemolyticus*; hi*Hh*, haemin-independent *H. haemolyticus*.

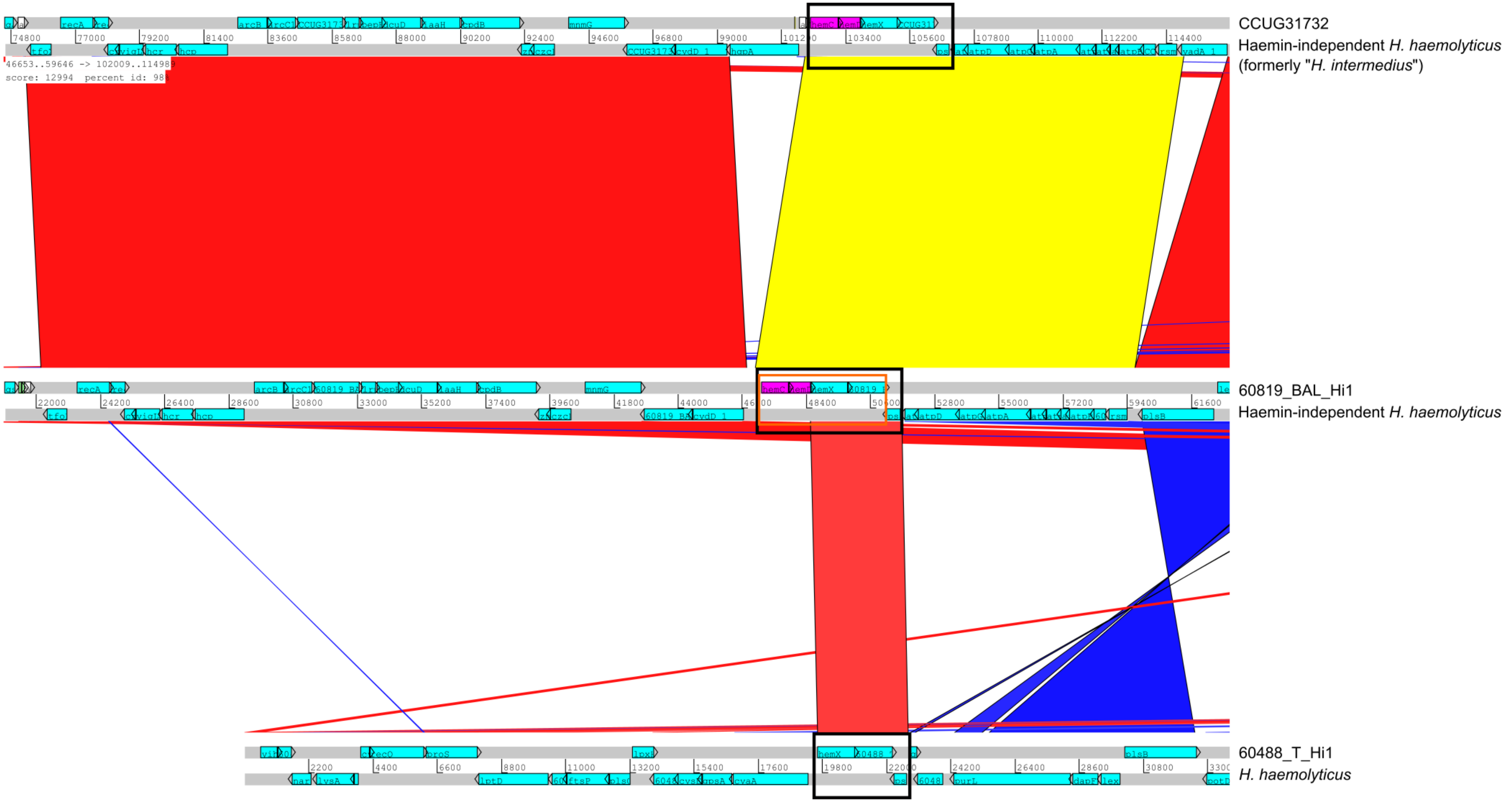

Figure S3. Artemis comparison tool alignment of two haemin-independent *Haemophilus haemolyticus* (hi*Hh*) and a haemin-dependent *H. haemolyticus* for visualisation of the syntenic block harbouring *hemC* and *hemD* (black box) predicted by progressiveMAUVE (54). Strain

names and their haemin-dependence are noted beside the respective genome. Coding sequences are indicated by teal blocks containing the PROKKA (45) gene annotation, except for *hemC* and *hemD* (pink). Nucleotide sequence shared between the hi*Hh* strain 60819_BAL_Hi1 genome and the other two genomes are signified by red (same orientation) or blue (inverted) blocks. The yellow block encompasses the *hemC* and *hemD* genes. The orange box indicates sequence orientation surrounding *hemCD* shared between hi*Hh* and *H. parainfluenzae*.

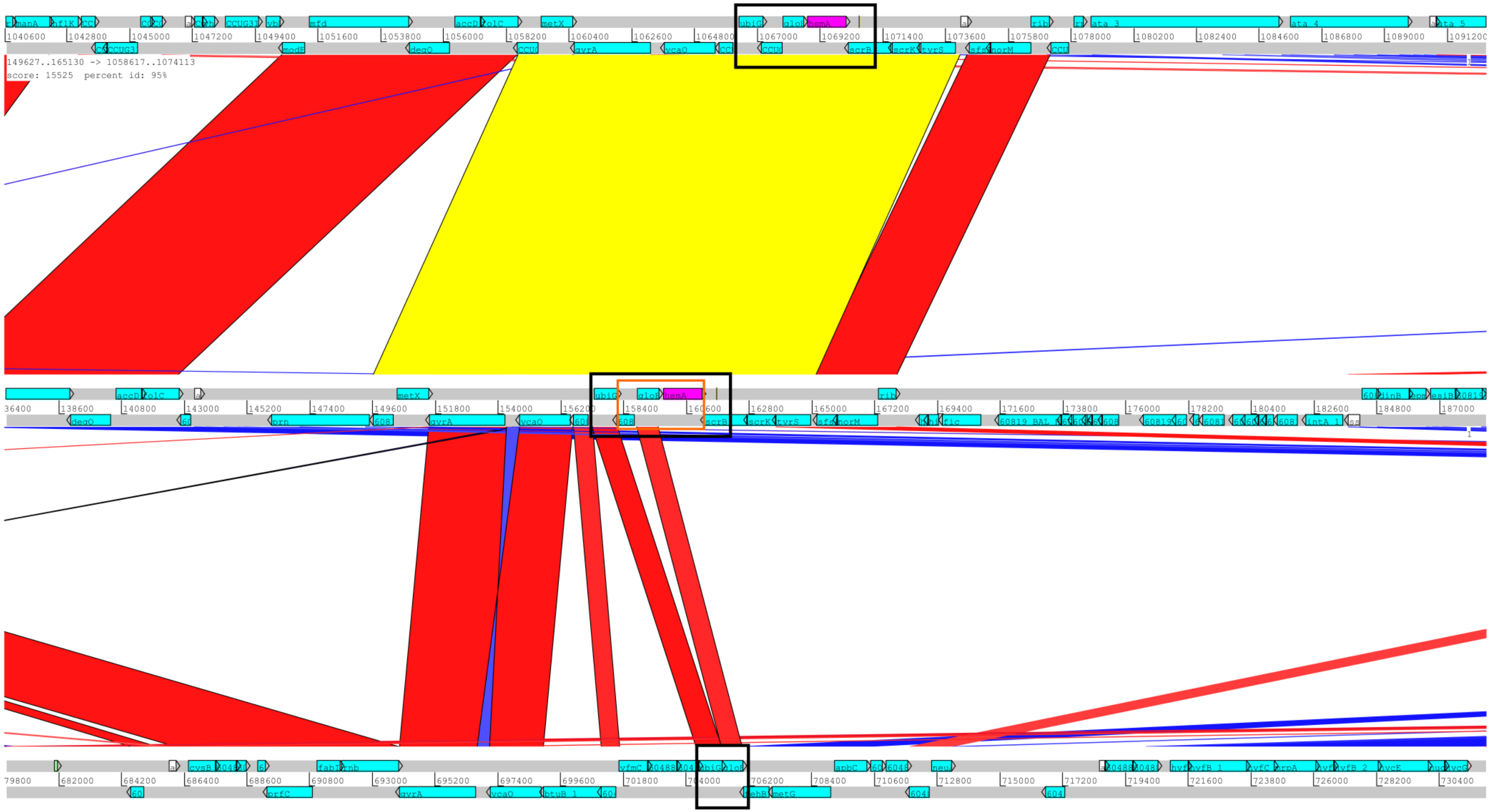

Figure S4. Artemis comparison tool alignment of two haemin-independent *Haemophilus haemolyticus* (hi*Hh*) and a haemin-dependent *H. haemolyticus* for visualisation of the *hemA* syntenic block (black box) predicted by progressiveMAUVE (54). Depicted genomes correspond

to the genomes in Figure S3. Coding sequences are indicated by teal blocks containing the PROKKA (45) gene annotation, except for *hemA* (pink). Nucleotide sequence shared between the hi*Hh* strain 60819_BAL_Hi1 genome and the other two genomes are signified by red (same orientation) or blue (inverted) blocks. The yellow block encompasses the *hemA* gene. The orange box indicates sequence orientation surrounding *hemA* shared between hi*Hh* and *H. parainfluenzae*.

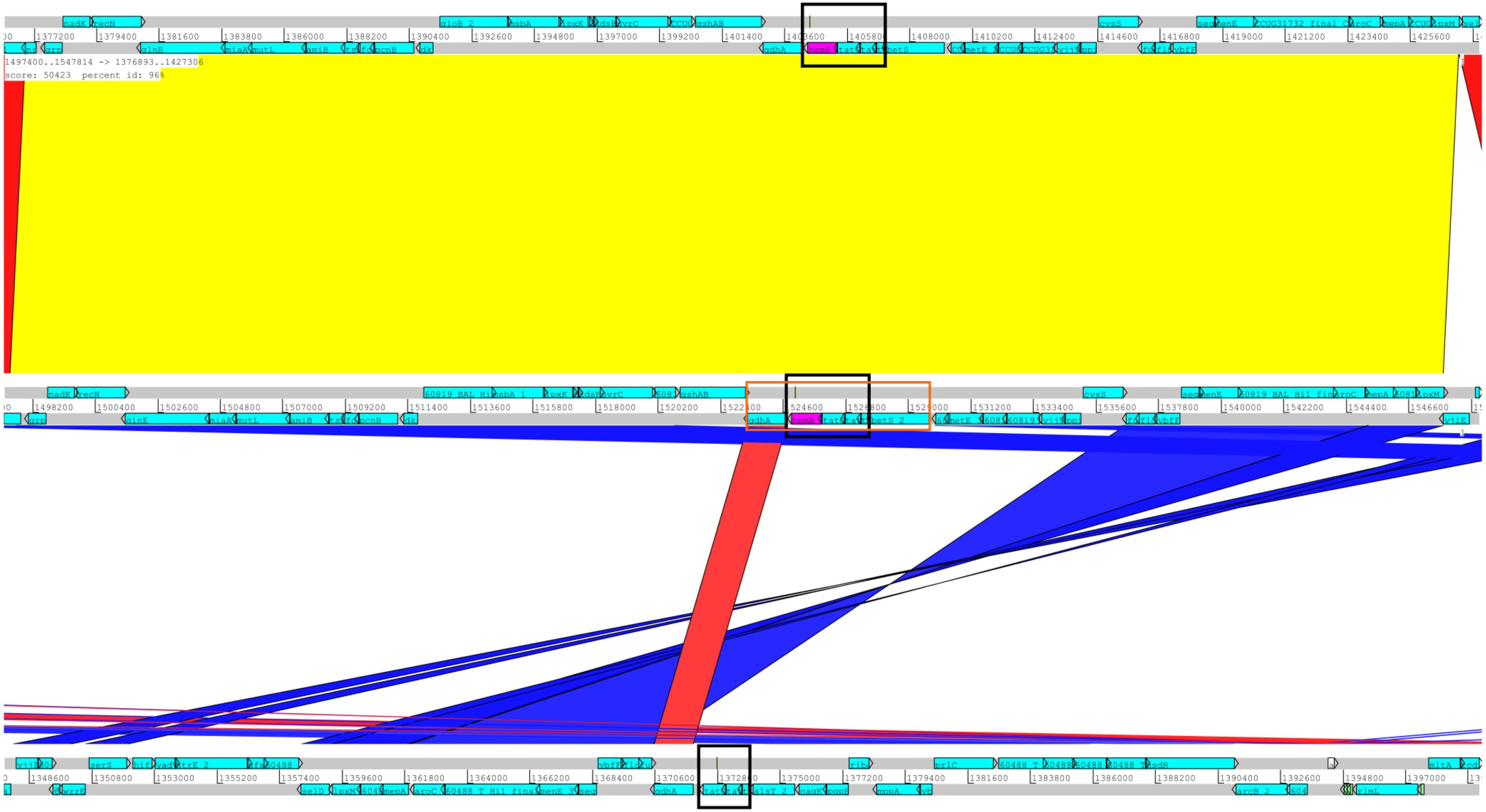

Figure S5. Artemis comparison tool alignment of two haemin-independent *Haemophilus haemolyticus* (hi*Hh*) and a haemin-dependent *H. haemolyticus* for visualisation of the *hemB* syntenic block (black box) predicted by progressiveMAUVE (54). Depicted genomes

correspond to the genomes in Figure S3. Coding sequences are indicated by teal blocks containing the PROKKA (45) gene annotation, except for *hemB* (pink). Nucleotide sequence shared between the hi*Hh* strain 60819_BAL_Hi1 genome and the other two genomes are signified by red (same orientation) or blue (inverted) blocks. The yellow block encompasses the *hemB* gene. The orange box indicates sequence orientation surrounding *hemB* shared between hi*Hh* and *H. parainfluenzae*.

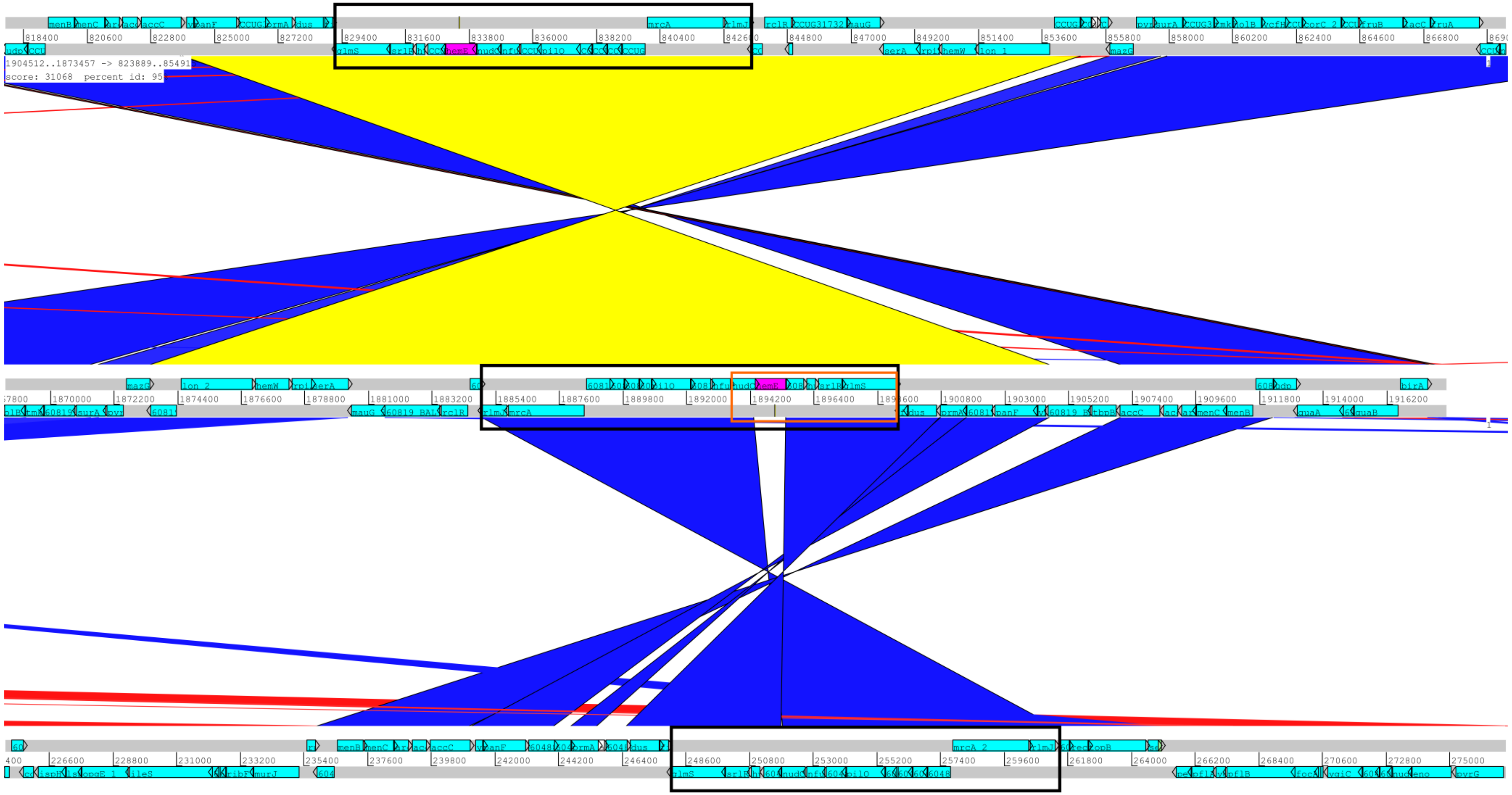

Figure S6. Artemis comparison tool alignment of two haemin-independent *Haemophilus haemolyticus* (hi*Hh*) and a haemin-dependent *H. haemolyticus* for visualisation of the *hemE* syntenic block (black box) predicted by progressiveMAUVE (54). Depicted genomes correspond

to the genomes in Figure S3. Coding sequences are indicated by teal blocks containing the PROKKA (45) gene annotation, except for *hemE* (pink). Nucleotide sequence shared between the hi*Hh* strain 60819_BAL_Hi1 genome and the other two genomes are signified by red (same orientation) or blue (inverted) blocks. The yellow block encompasses the *hemE* gene. The orange box indicates sequence orientation surrounding *hemE* shared between hi*Hh* and *H. parainfluenzae*.

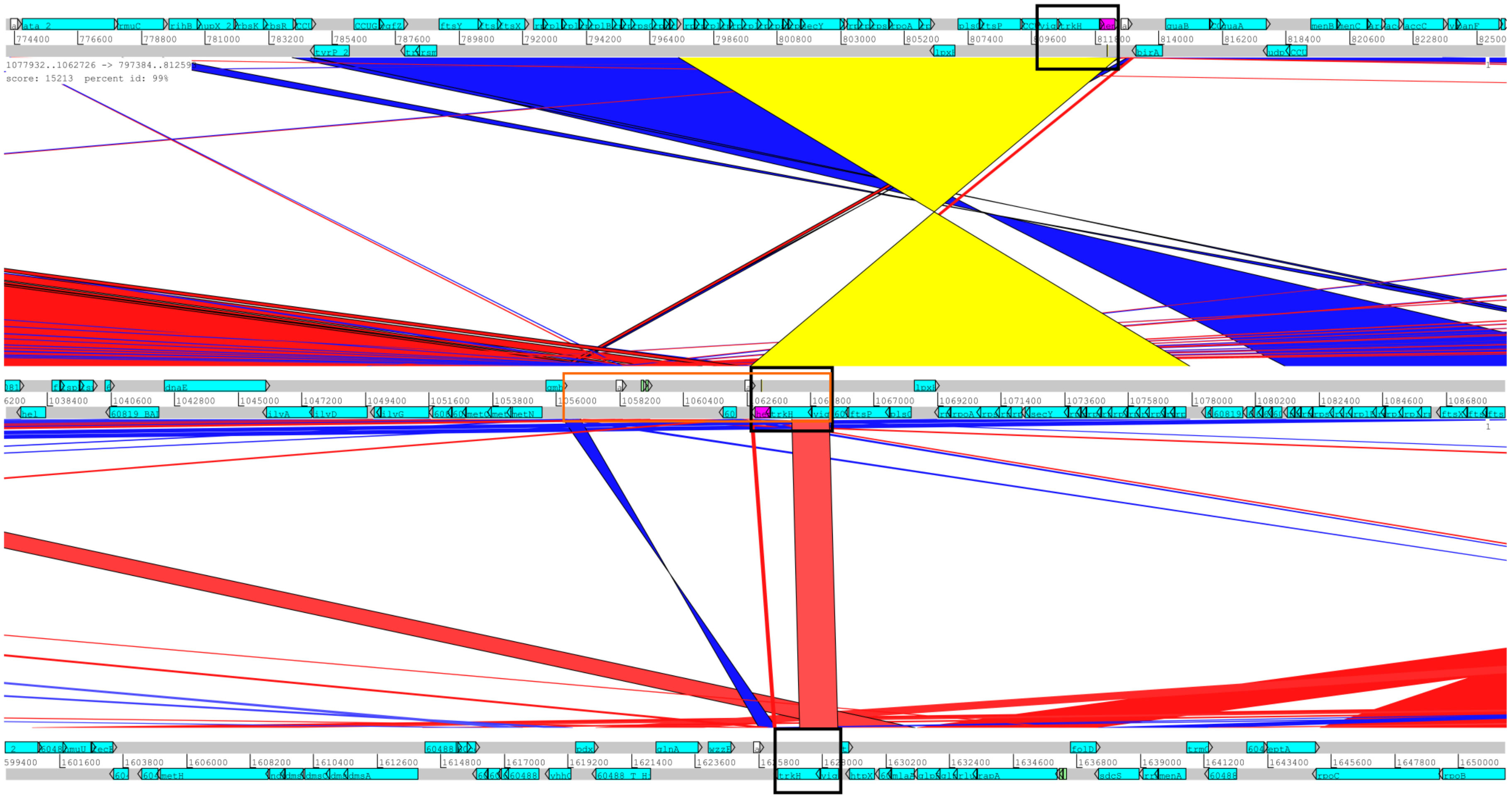

Figure S7. Artemis comparison tool alignment of two haemin-independent *Haemophilus haemolyticus* (hi*Hh*) and a haemin-dependent *H. haemolyticus* for visualisation of the *hemG* syntenic block (black box) predicted by progressiveMAUVE (54). Depicted genomes correspond

to the genomes in Figure S3. Coding sequences are indicated by teal blocks containing the PROKKA (45) gene annotation, except for *hemG* (pink). Nucleotide sequence shared between the hi*Hh* strain 60819_BAL_Hi1 genome and the other two genomes are signified by red (same orientation) or blue (inverted) blocks. The yellow block encompasses the *hemG* gene. The orange box indicates sequence orientation surrounding *hemG* shared between hi*Hh* and *H. parainfluenzae*.

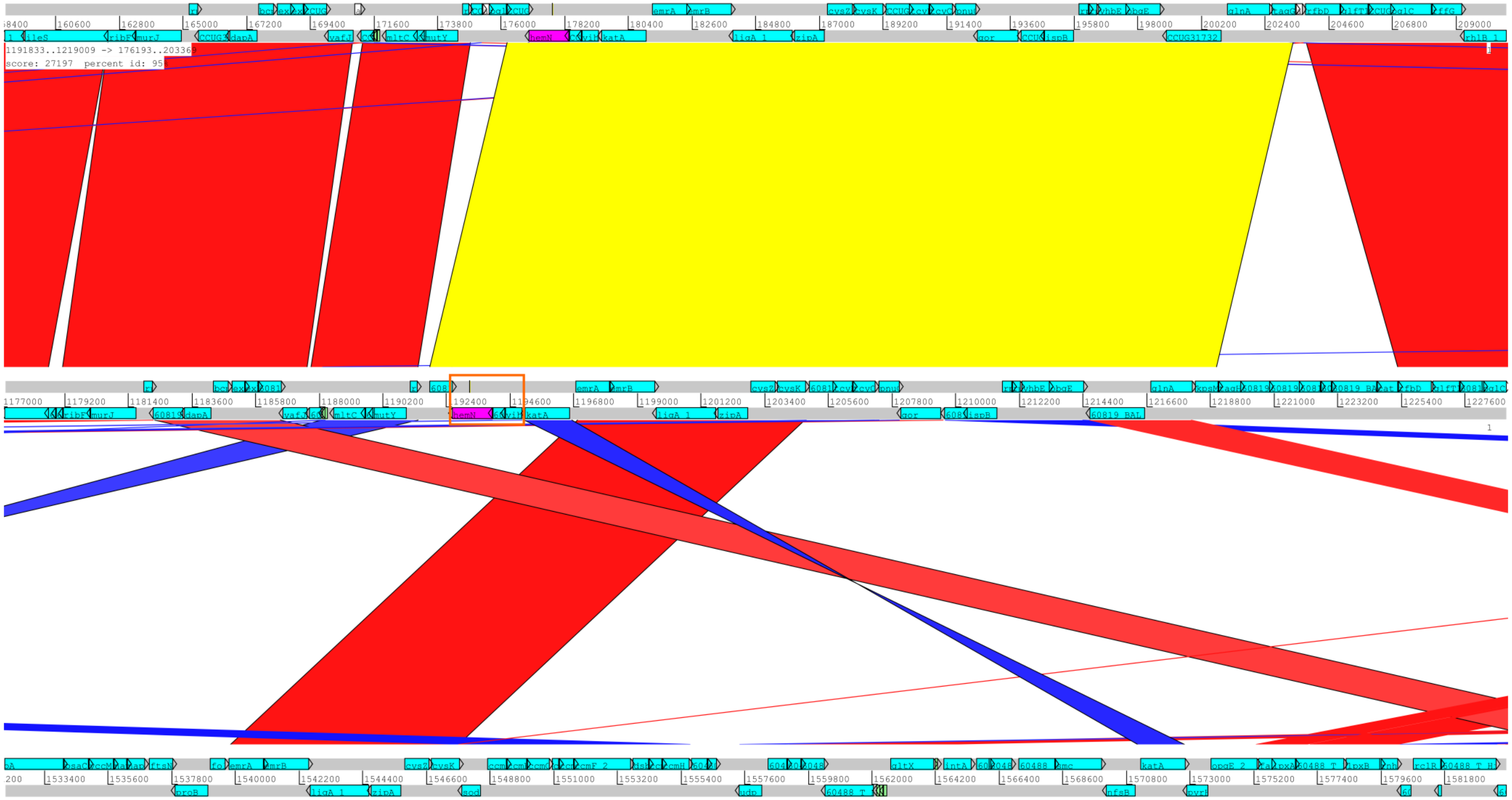

Figure S8. Artemis comparison tool alignment of two haemin-independent *Haemophilus haemolyticus* (hi*Hh*) and a haemin-dependent *H. haemolyticus* for visualisation of the genomic region that encompasses *hemN*. Depicted genomes correspond to the genomes in Figure S3.

Coding sequences are indicated by teal blocks containing the PROKKA (45) gene annotation, except for *hemN* (pink). Nucleotide sequence shared between the hi*Hh* strain 60819_BAL_Hi1 genome and the other two genomes are signified by red (same orientation) or blue (inverted) blocks. The yellow block encompasses the *hemN* gene. The orange box indicates sequence orientation surrounding *hemN* shared between hi*Hh* and *H. parainfluenzae*.

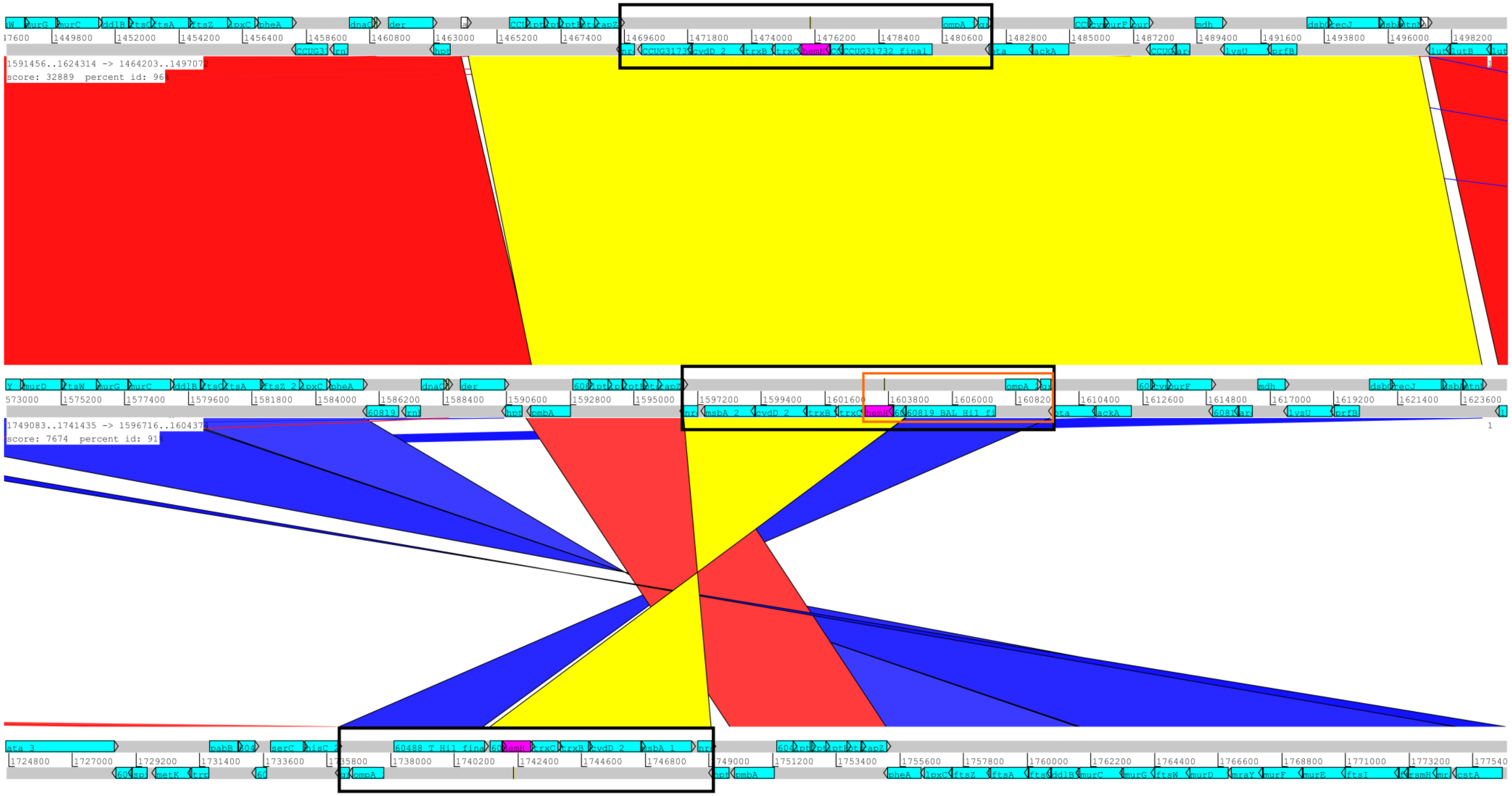

Figure S9. Artemis comparison tool alignment of two haemin-independent *Haemophilus haemolyticus* (hi*Hh*) and a haemin-dependent *H. haemolyticus* for visualisation of the *hemH* syntenic block (black box) predicted by progressiveMAUVE (54). Depicted genomes correspond

to the genomes in Figure S3. Coding sequences are indicated by teal blocks containing the PROKKA (45) gene annotation, except for *hemH* (pink). Nucleotide sequence shared between the hi*Hh* strain 60819_BAL_Hi1 genome and the other two genomes are signified by red (same orientation) or blue (inverted) blocks. The yellow blocks encompass the *hemH* gene. The orange box indicates sequence orientation surrounding *hemH* shared between hi*Hh* and *H. parainfluenzae*.

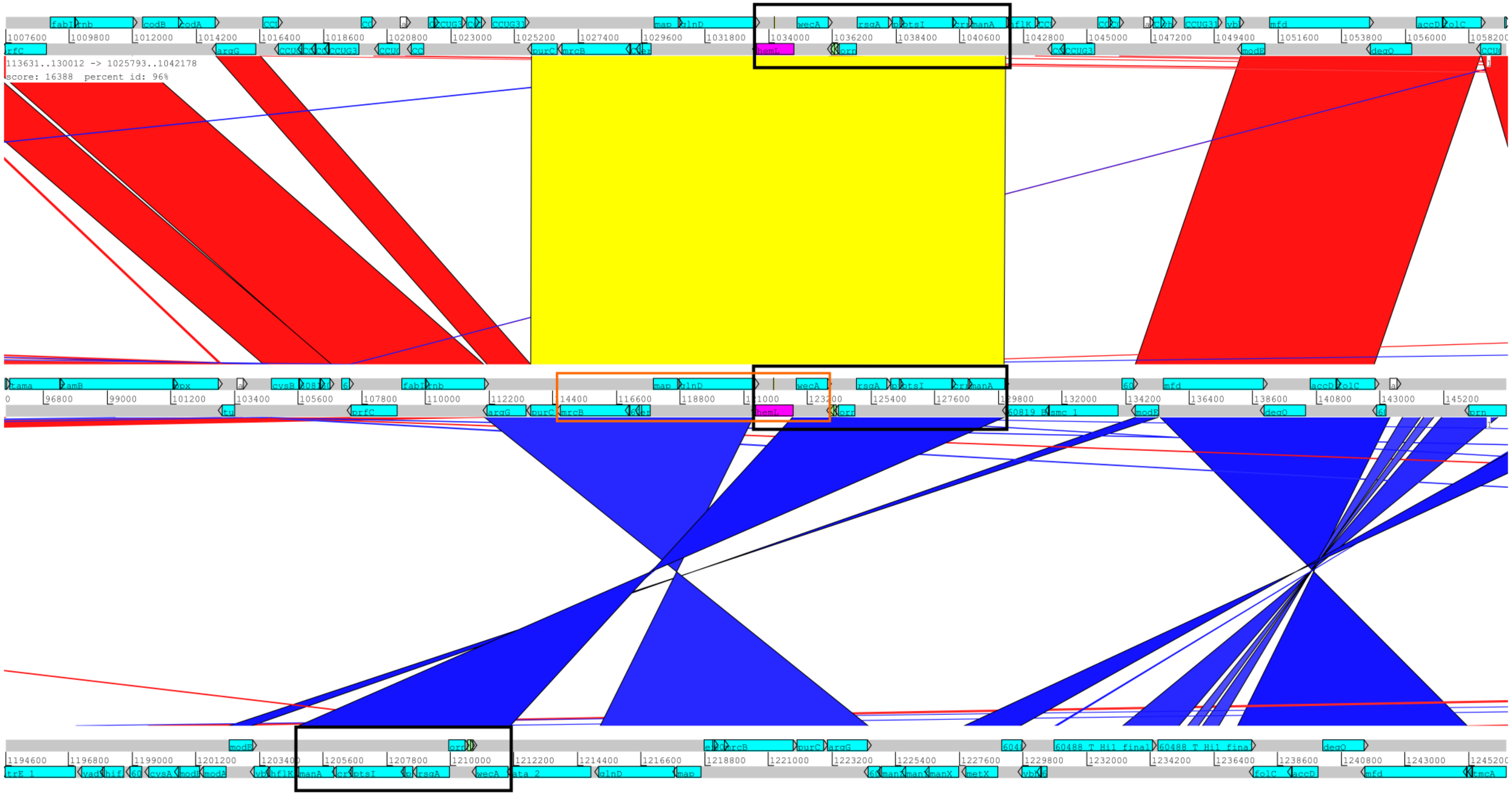

Figure S10. Artemis comparison tool alignment of two haemin-independent *Haemophilus haemolyticus* (hi*Hh*) and a haemin-dependent *H. haemolyticus* for visualisation of the *hemL* syntenic block (black box) predicted by progressiveMAUVE (54). Depicted genomes

correspond to the genomes in Figure S3. Coding sequences are indicated by teal blocks containing the PROKKA (45) gene annotation, except for *hemL* (pink). Nucleotide sequence shared between the hi*Hh* strain 60819_BAL_Hi1 genome and the other two genomes are signified by red (same orientation) or blue (inverted) blocks. The yellow block encompasses the *hemL* gene. The orange box indicates sequence orientation surrounding *hemL* shared between hi*Hh* and *H. parainfluenzae*.
